# New insights into the anti-inflammatory and anti-melanoma mechanisms of action of azelaic acid and other *Fusarium solani* metabolites via in vitro and in silico studies

**DOI:** 10.1038/s41598-024-63958-0

**Published:** 2024-06-22

**Authors:** Mona Ismail, Marwa H. A. Hassan, Enas I. A. Mohamed, Ahmed F. Azmy, Abeer Moawad, Rabab Mohammed, Mohamed A. Zaki

**Affiliations:** 1 Department of Pharmacognosy, Faculty of Pharmacy, Beni-Suef University, Beni-Suef, 62514 Egypt; 2 Department of Microbiology and Immunology, Faculty of Pharmacy, Beni-Suef University, Beni-Suef, 62514 Egypt

**Keywords:** *Fusarium solani*, *Euphorbia tirucalli*, Cyclooxygenase (COX-1 and COX-2), Docking, Melanoma, Pirin, Azelaic, Computational biology and bioinformatics, Drug discovery

## Abstract

Metabolites exploration of the ethyl acetate extract of *Fusarium solani* culture broth that was isolated from *Euphorbia tirucalli* root afforded five compounds; 4-hydroxybenzaldehyde (**1**), 4-hydroxybenzoic acid (**2**), tyrosol (**3**), azelaic acid (**4**), malic acid (**5**), and fusaric acid (**6**). Fungal extract as well as its metabolites were evaluated for their anti-inflammatory and anti-hyperpigmentation potential via in vitro cyclooxygenases and tyrosinase inhibition assays, respectively. Azelaic acid (**4**) exhibited powerful and selective COX-2 inhibition followed by fusaric acid (**6**) with IC_50_ values (2.21 ± 0.06 and 4.81 ± 0.14 μM, respectively). As well, azelaic acid (**4**) had the most impressive tyrosinase inhibitory effect with IC_50_ value of 8.75 ± 0.18 μM compared to kojic acid (IC_50_ = 9.27 ± 0.19 μM). Exclusive computational studies of azelaic acid and fusaric acid with COX-2 were in good accord with the in vitro results. Interestingly, this is the first time to investigate and report the potential of compounds **3**–**6** to inhibit cyclooxygenase enzymes. One of the most invasive forms of skin cancer is melanoma, a molecular docking study using a set of enzymes related to melanoma suggested pirin to be therapeutic target for azelaic acid and fusaric acid as a plausible mechanism for their anti-melanoma activity.

## Introduction

Natural products are usually a promising source for active drugs. One of the recently promising sources of natural products is endophytes. Endophytes are microorganisms that can survive inter- or intracellularly in plants for at least a portion of their lives without inflecting outward signs of infection. In many cases, endophytes afford new active natural products, while in some cases they have the potential to synthesize similar products to those yielded by the host plants^1^. Although *Fusarium* species have been considered for many years as significant plant pathogens and as one of the main manufacturers of mycotoxins, such as fumonisin, zearalenone, and trichothecene, that can lead to diseases in humans, plants and animals^2,3^, *Fusarium* species have been identified as a significant source of various constituents with diverse therapeutic values such as pyranones, alkaloids, amides, quinones, peptides, and terpenoids^4^. Herein, we investigated the endophytic fungus *Fusarium solani*, which was cultured from the roots of *Euphorbia tirucalli*. *Euphorbia tirucalli is* a subtropical and tropical ornamental succulent cactus-like plant that is often known as aveloz or pencil tree and is used in folk medicine to treat many types of cancer, such as prostate cancer, basal cell carcinoma, breast cancer, and leukemia^5^, it has been reported to have antibacterial^6^, analgesic, anti-inflammatory^7^, hepato-protective, antioxidant^8^, cytotoxic^9^ and antimicrobial activities^10^. Our previous exploration of the phytoconstituents of the aerial parts of *Euphorbia pseudocactus* led to the isolation and identification of astragalin, kaempferol, nicotiflorin, astragalin-6′′-gallate, gallic acid, ethyl gallate, 1,2,3,4,6-pentagalloylglucose, and ellagic acid, further biological investigation unveiled that gallic acid exhibited good anticancer activity against two colorectal cell lines: LS-174T and LS-513^11^.

Large-scale fermentation of the isolated fungus over Sabouraud Dextrose Broth media (SDB) led to separation and identification of six natural metabolites (**1**–**6**); 4-hydroxybenzaldehyde (**1**), 4-hydroxybenzoic acid (**2**), tyrosol (**3**), azelaic acid (**4**), malic acid (**5**), and fusaric acid (**6**). Structural elucidation of the isolated metabolites was based on different spectroscopic techniques. Some of the isolated metabolites have been reported to possess effects on skin including wound healing, hyperpigmentation disorders, and melanoma^12–18^. Since inflammation is a common feature in all those conditions, therefore cyclooxygenase along with tyrosinase inhibitory activities of the fungal extract and the isolated metabolites have been carried out to study their anti-inflammatory and anti-hyperpigmentation potential. A molecular docking experiment has been performed to elucidate mode of interaction with cyclooxygenase enzymes.

Azelaic acid (**4**) has been reported to possess diverse biological activities and to be used in management of cutaneous and hyper-pigmentary disorders as well as melanoma^19,20^. 4-hydroxybenzoic acid was found active against certain human and murine melanoma^21^ and fusaric acid as well unveiled cytotoxicity against melanoma cell lines^22^. Although previous research attributed the anti-melanoma activity of azelaic acid to the inhibition of thioredoxin reductase activity and DNA synthesis in melanoma cells, its mechanism of action is still ambiguous^23–25^, for this reason, a molecular docking study was conducted to understand the anti-melanoma mechanism of action of azelaic acid along with the other isolated metabolites via evaluation of their potential binding against a set of enzymes related to melanoma to discern which of them may be targeted by the metabolites. Results of the molecular docking study shed light on pirin as a possible target for the anti-melanoma activity of azelaic acid. Pirin is an iron-containing nuclear protein and transcription cofactor that is conserved to a large extent between different organisms^26^. It is detected in normal tissue in low concentrations, while it is detected at elevated levels in cancerous cells especially melanomas^27^. Until recently, its exact function was unrecognized, Miyazaki and co-workers reported that knockdown or inhibition of pirin resulted in inhibition of the migration of melanoma cells and they discovered an inhibitor that binds to a small molecule-binding pocket in pirin^26^.

## Results and discussion

### Morphological identification of the endophytic fungus

**Figure 1:**
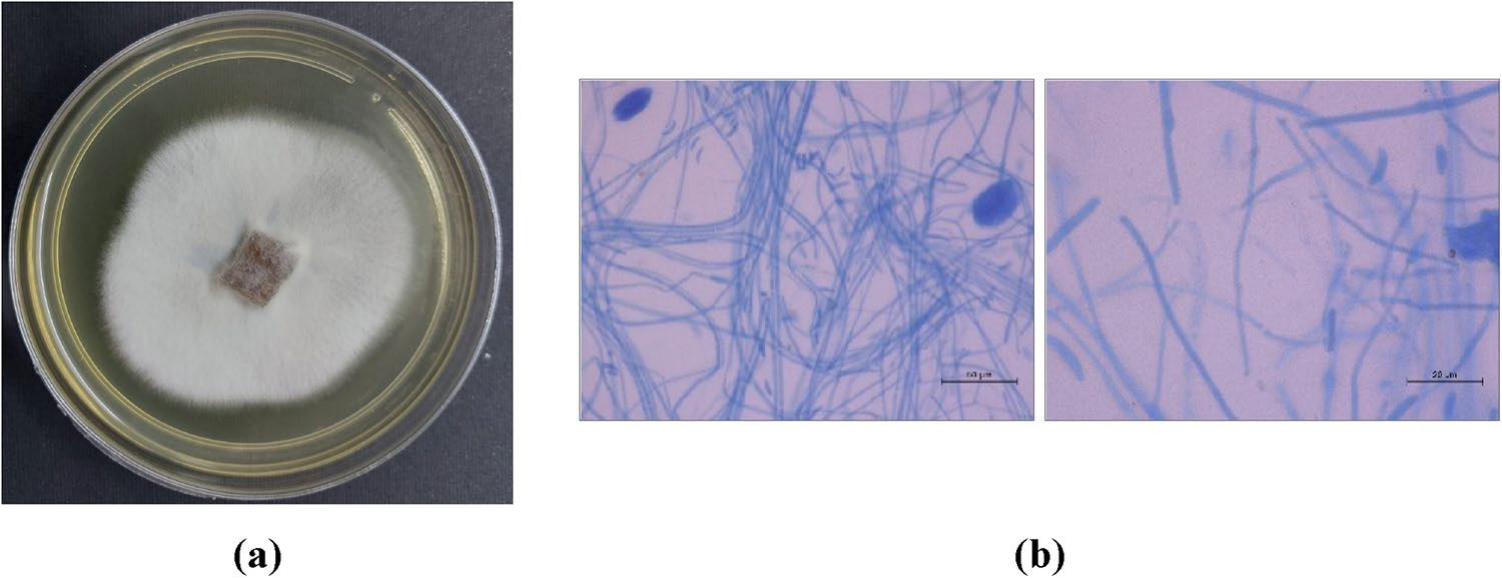
The fungus morphology. (**a**) Morphological characteristics of mycelial growth on SDA medium, (**b**) the morphology of spore and hyphae stained with Lactophenol cotton blue stain.

The roots of the *E. tirucalli* plant produced different fungal colonies when cultured on SDA medium, the fungal colonies were further purified using successive culture on SDA plates. Colonies with white cottony appearance were chosen for further microscopic identification. Microscopic analysis showing curved microconidia with rounded ends were primarily identified as *Fusarium* species. The fungus morphology (Fig. 1).

### Molecular identification of the endophytic fungus

**Figure 2:**
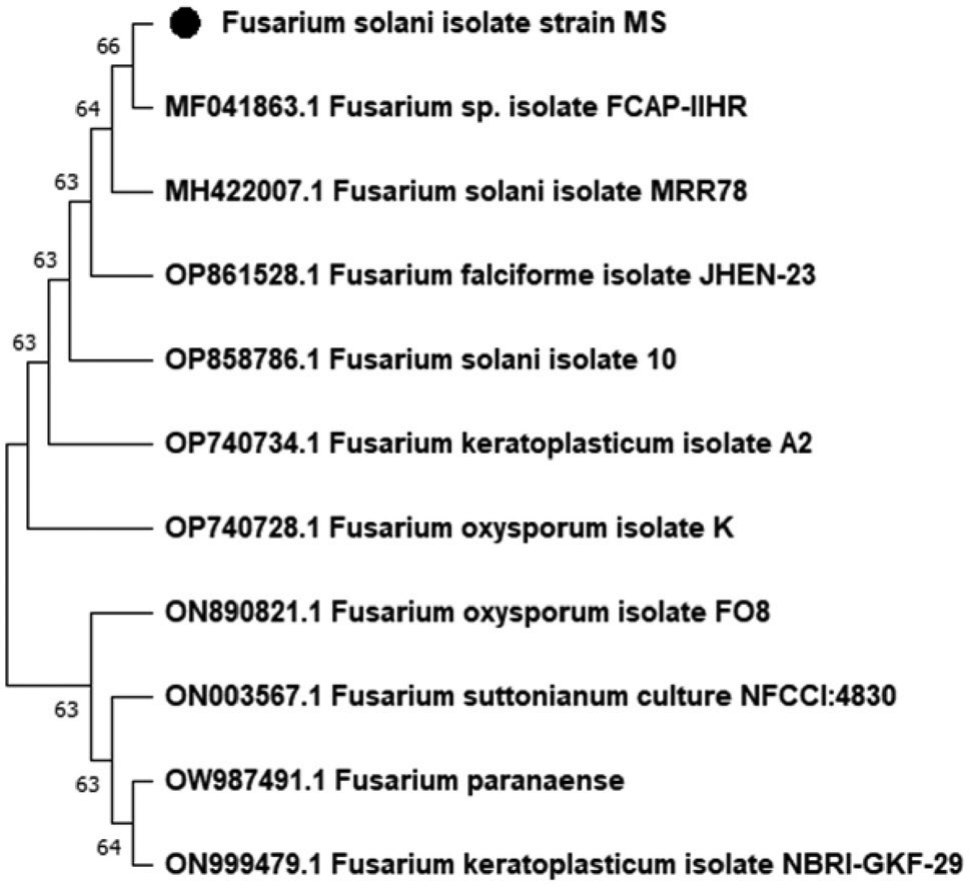
The evolutionary history was inferred using the Neighbor-Joining method ^67^. The bootstrap consensus tree inferred from 500 replicates ^68^ is taken to represent the evolutionary history of isolated *Fusarium solani* ^68^. Branches corresponding to partitions reproduced in less than 50% bootstrap replicates are collapsed. The percentage of replicate trees in which the associated taxa clustered together in the bootstrap test (500 replicates) are shown next to the branches ^68^. The evolutionary distances were computed using the Kimura 2-parameter method ^69^ and are in the units of the number of base substitutions per site. All positions containing gaps and missing data were eliminated (complete deletion option). Evolutionary analyses were conducted in MEGA11 ^65^.

Blast analysis of the resulting ITS (Internal transcribed spacer) gene sequence showed 100% similarity with *Fusarium solani* isolate, using the following primers, forward:0.5′-TCCTCCGCTTATTGATATGC-3 and reverse: 5′ GGAAGTAAAAGTCGTAACAAGG-3 ^28^. The *Fusarium solani* morphology (Fig. 1) and the phylogenetic tree (Fig. 2) revealed the powerful relation between this isolate and other *Fusarium spp.* mainly *Fusarium solani*.

### Identification of the isolated compounds

**Figure 3:**
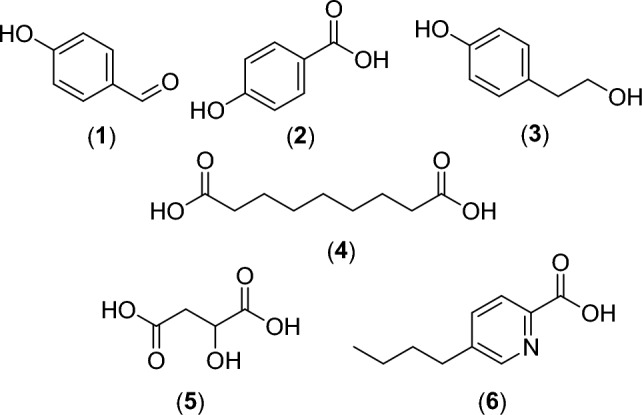
Structures of the isolated metabolites (**1**–**6**) from the endophytic fungus *Fusarium solani*.

Identification and structural elucidation of the isolated metabolites (**1**–**6**) were based on NMR (Nuclear Magnetic Resonance) spectral data (Supplementary data: Figure S1–S12) and comparison of spectral data with those previously published. The isolated compounds (**1**–**5**) were previously isolated from *F. solani*^2,29,30^, while compound (**6**) has been isolated for the first time from *F. solani*. The isolated compounds (Fig. 3) were identified as 4-hydroxybenzaldehyde (**1**)^29^, 4-hydroxybenzoic acid (**2**)^2,31^, tyrosol (**3**)^30,32^, azelaic acid (**4**)^2,33^, malic acid (**5**)^2,34^, and fusaric acid (**6**)^35^. Benzoic acid and malic acid (**5**) were previously isolated from the host plant *E. tirucalli*^36,37^, while benzoic acid derivative; 4-hydroxybenzoic acid (**2**) and malic acid (**5**) were formerly isolated from the endophytic fungus *F. solani*^2^ which indicates that the endophytic microbe would be able to produce the same or comparable substances to those produced by the host plant.

### COX-1 and COX-2 inhibition assay

Cyclooxygenase enzymes are oxidoreductases which induce the dioxygenation of arachidonic acid into prostaglandin H_2_ that is transformed to a number of signaling molecules involved in inflammation process. The anti-inflammatory capability of the extract and the isolated metabolites (**1**–**6**) were estimated via in vitro inhibition of COX-1 and COX-2. The COX-1 and COX-2 inhibitory study results (Table 1) revealed that the extract moderately inhibits COX-1 and COX-2 enzymes with (IC_50_ = 63.88 ± 0.59 and 12.21 ± 0.36 μg/ml), respectivly. While azelaic acid (**4**) demonstrated the most potent and selective COX-2 inhibition activity (IC_50_ = 2.21 ± 0.06 μM, SI = 5.24) compared to the standard inhibitor celecoxib (IC_50_ = 0.82 ± 0.02 μM, SI = 18.3) followed by fusaric acid (**6**), tyrosol (**3**), 4-hydroxybenzaldehyde (**1**), malic acid (**5**), and 4-hydroxybenzoic acid (**2**) with IC_50_ values of 4.81 ± 0.14, 5.31 ± 0.15, 8.87 ± 0.26, 10.41 ± 0.30, and 28.44 ± 0.76 μM, respectively and selectivity indices of 0.80, 4.49, 2.90, 4.90, and 1.65, respectively. Regarding inhibitory activity against COX-1; fusaric acid (**6**) and azelaic acid (**4**) showed the highest inhibition for COX-1 with IC_50_ values 3.87 ± 0.05 and 11.62 ± 0.29 μM, respectively. Previous studies have already indicated that azelaic acid (**4**) exhibited anti-inflammatory effect on normal human keratinocytes through suppression of the interleukins (IL-1, IL-6, and IL-8) and tumor necrosis factor (TNF)-α^38^ and that tyrosol (**3**) showed anti-inflammatory effect on human endothelial cells^39^ also malic acid has been reported to reduce (TNF)-α^40^ and to suppress gout combined inflammation in animal models^41^ but this is the first time to investigate and report the potential of tyrosol, azelaic acid, malic acid, and fusaric acid to inhibit cyclooxygenase enzymes and our results unravel an additional mechanism by which azelaic acid exerts its anti-inflammatory activity and provide further evidence for its efficacy against inflammatory skin diseases.

**Table 1:** IC_50_ and selectivity index of the total extract and the isolated metabolites from the endophytic fungus *Fusarium solani* against COX-1 and COX-2 enzymes.

Extract/compounds	IC50 (μM)		Selectivity index (SI)
	COX-1	COX-2	
Extract	63.88 ± 0.59*^a^	12.21 ± 0.36*^b^	5.23
4-Hydroxybenzaldehyde (**1**)	25.72 ± 0.55^a^	8.87 ± 0.26^b^	2.9
4-Hydroxybenzoic acid (**2**)	44.50 ± 0.92^a^	28.44 ± 0.76^b^	1.65
Tyrosol (**3**)	23.86 ± 0.38^a^	5.31 ± 0.15^b^	4.49
Azelaic acid (**4**)	11.62 ± 0.29^a^	2.21 ± 0.06^b^	5.24
Malic acid (**5**)	51.02 ± 0.86^a^	10.41 ± 0.30^b^	4.9
Fusaric acid (**6**)	3.86 ± 0.05^a^	4.81 ± 0.14^b^	0.8
Indomethacin	0.28 ± 0.006	0.46 ± 0.01	0.6
Celecoxib	15.18 ± 0.38	0.82 ± 0.02	18.3

Data represented in the table as mean ± standard error of the mean (SEM), ^a^Significantly different from Indomethacin and ^b^Significantly different from Celecoxib at *p* < 0.05, *IC_50_ is expressed as µg/ml.

### Tyrosinase inhibitory assay

The effectiveness of the total extract and the isolated compounds (**1**–**6**) to inhibit tyrosinase enzyme was detemined, compared to kojic acid as a reference drug. The results demonstrated in (Table 2) showed that azelaic acid had considerable tyrosinase inhibitory effect with IC_50_ value of 8.75 ± 0.18 μM which was comparable with that of kojic acid (IC_50_ = 9.27 ± 0.19 μM). Tyrosol showed inhibitory activity with IC_50_ of 11.35 ± 0.24 μM, followed by fusaric acid with IC_50_ of 13.42 ± 0.26 μM. The total extract of the endophytic fungus and 4-hydroxybenzoic acid showed moderate inhibitory effect with IC_50_ of 21.79 ± 0.44 and 25.45 ± 0.54 μM, respectively in comparison with the previous compounds. On the other hand, 4-hydroxybenzaldehyde and malic acid showed IC_50_ > 50 μM indicating very little inhibitory effect. All mammalian melanocytes contain the tyrosinase enzyme, which limits the rate at which melanin is produced^42^. Tyrosinase is over expressed by aggressive tumors such as melanoma, so, it is necessary to control tyrosinase activity in order to avoid the excess production of melanin^43^.

**Table 2:** IC_50_ of tyrosinase inhibitory activity of the total extract and the isolated metabolites from the endophytic fungus *Fusarium solani.*

Extract/compounds	IC_50_ (μM)
Extract	21.79 ± 0.44*^a^
4-hydroxybenzaldehyde (**1**)	70.60 ± 1.49^a^
4-hydroxybenzoic acid (**2**)	25.45 ± 0.53^a^
Tyrosol (**3**)	11.35 ± 0.24
Azelaic acid (**4**)	8.75 ± 0.18
Malic acid (**5**)	87.46 ± 1.84^a^
Fusaric acid (**6**)	13.42 ± 0.27
Kojic acid	9.27 ± 0.19

Data represented in the table as mean ± standard error of the mean (SEM), ^a^Significantly different from Kojic acid at *p* < 0.05, *IC_50_ is expressed as µg/ml.

### Molecular docking study

**Figure 4:**
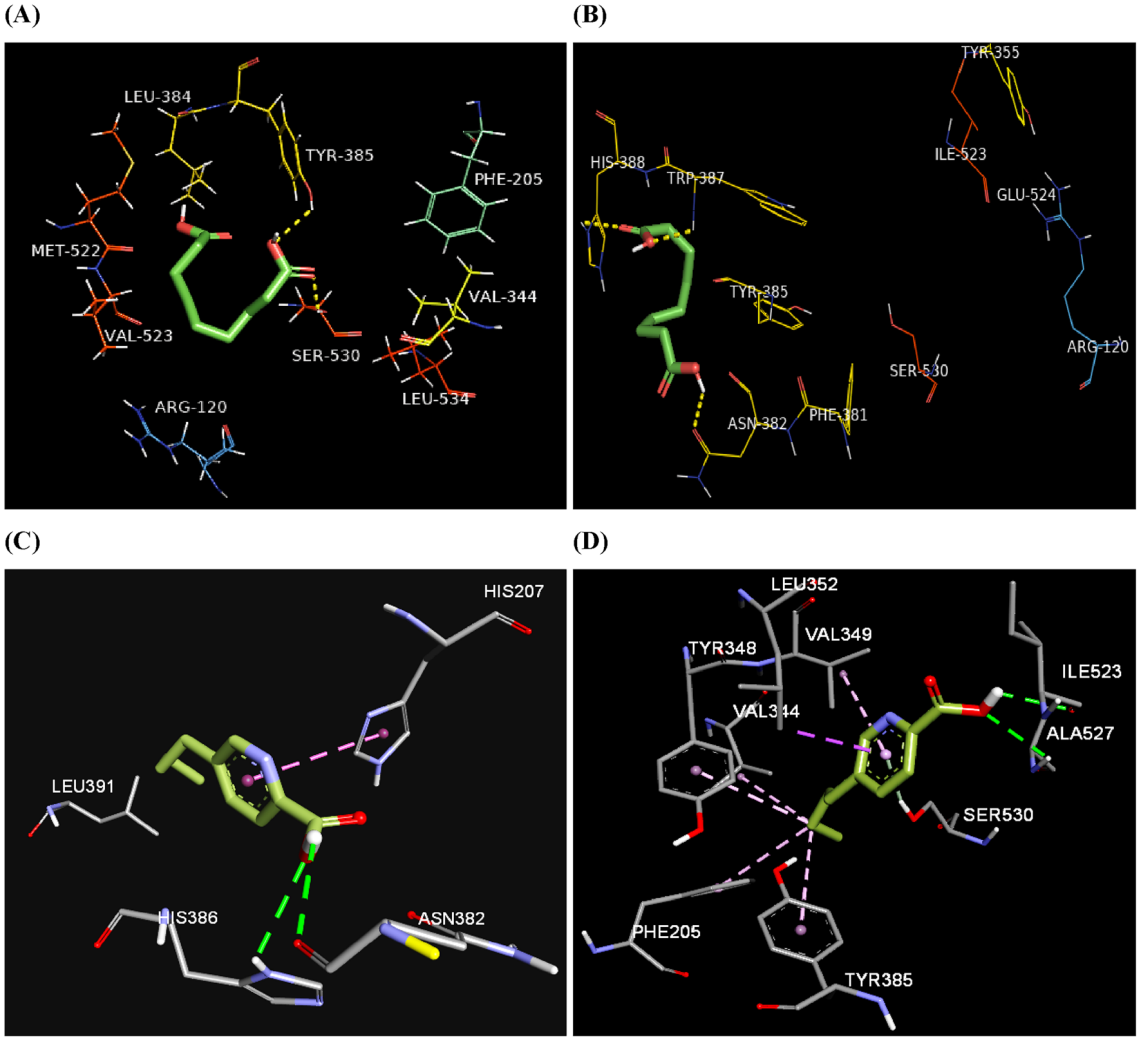
Binding modes of azelaic acid (**A**), (**B**) and fusaric acid (**C**), (**D**) into COX-2 (5IKV) and COX-1 (6Y3C) binding sites, respectively. Ligands are depicted in green tube models. The amino acids are shown in labelled line models. Hydrogen bonds are represented by yellow and green dashed lines, while hydrophobic interactions by pink and magenta dashed lines.

**Figure 5:**
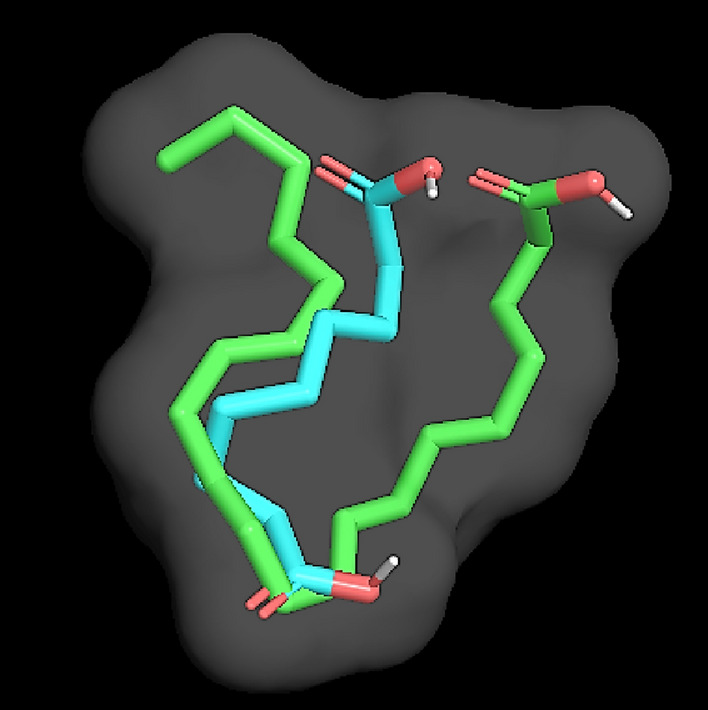
Superposition of arachidonic acid (green) and azelaic acid (blue) bound in the active site of COX-2 indicating similar conformation.

For more understanding of the COX inhibition activity at the molecular level, a computational study has been conducted to investigate binding of the metabolites into the active sites of COX enzymes. Cyclooxygenases are oxidoreductase enzymes of two isoforms COX-1 and COX-2. Both isoforms are homodimer enzymes of three domains, where they are similar to a great extent in their amino acids sequence^44,45^. The entrance to the active site cavity of COX-2 is surrounded by three amino acids Arg120, Tyr355, and Glu524 and afterwards, the binding site is located near the catalytically significant Tyr385 and comprised the amino acids Val523, Val434, Arg513, and Leu503^46^. In COX-1, substitution of Val523 with the sterically hindered Ile523 causes the presence of an additional side pocket in COX-2 allowing larger active site^47^. The docking study was conducted exclusively on the COX enzymes for azelaic and fusaric acids since these two compounds have not been docked into the active site of COXs previously. Binding affinities and intermolecular interactions are summarized in (Table 3). Docking of azelaic acid into binding site of COX-2 revealed hydrogen bond with Ser530 located between Arg120 at the entrance of the enzyme channel and the buried Tyr385 in the hydrophobic pocket. This orientation of azelaic carboxylic oxygen to be in proximity to Ser530 hydroxyl group was supported by hydrogen bonding between the same carboxylic group and hydroxyl group of Tyr385. Moreover, the amino acids Leu352, Val523, and Ala527 showed alkyl-hydrophobic interactions with azelaic acid, while it exhibited van der Waals interactions with Phe205, Val344, Val349, Ser353, Phe381, Leu384, Tyr385, Trp387, Phe518, Met522, and Leu534 (Fig. 4A). The conformation resulted from docking of azelaic acid into active site of COX-2 is similar to that of the enzyme substrate arachidonic acid suggesting it may act as a competitive inhibitor (Fig. 5). It is worthy to note that docking of azelaic acid into COX-1 revealed hydrogen bonding with the buried amino acids Asn382, Trp387, and His388 but no interaction with Ser530 which is important for inhibition of the enzyme activity^48^. and this may explain the lower in vitro inhibition effect of azelaic acid against COX-1 (Fig. 4B). In silico analysis showed that fusaric acid in COX-2 formed two hydrogen bonds with Asn382 and π-π stacking with His207 in addition to alkyl-hydrophobic contacts with Ala199, Leu390, and Leu391 (Fig. 4C), while in COX-1; it depicted two hydrogen bonds with Ile523 and Ala527, π-donor hydrogen bond with Ser530, hydrophobic contacts with Leu352 (π-σ), Val349 (π-alkyl), and alkyl hydrophobic interactions with Phe205, Val344, Tyr348, and Tyr385 (Fig. 4D).

**Table 3:** Binding scores and comprehensive intermolecular interactions of the isolated metabolites from the endophytic fungus *Fusarium solani* and the targeted enzymes COX-2 and COX-1.

Ligand	COX-2			COX-1		
	Binding score (kcal/mol)	Interactions		Binding score (kcal/mol)	Interactions	
		H-bond	Hydrophobic		H-bond	Hydrophobic
4-hydroxybenzaldehyde (**1**)	− 5.9	Tyr385, Met522	Leu352, Ala527	− 5.7	Tyr385, Ile523, Ser530	Val349, Leu352, Ala527
4-hydroxybenzoic acid (**2**)	− 6.1	Thr206, Trp387	Ala202, Gln203	− 6.1	–	Val349, Leu352, Trp387
Tyrosol (**3**)	− 6.2	Gln203, Tyr385	ALa202	− 5.7	Val349	Leu352, Trp387
Azelaic acid (**4**)	− 5.7	Ser530, Tyr385	Leu352, Val523, Ala527, Phe205, Val344, Val349, Ser353, Phe381, Leu384, Tyr385, Trp387, Phe518, Met522, Leu534	− 5.8	Asn382, Trp387, His388	Ala202, Tyr385
Malic acid (**5**)	− 4.7	Ala378, Tyr385	Asn382	− 4.9	Thr206, Tyr385, His386, Trp387, His388	–
Fusaric acid (**6**)	− 6.7	Asn382	His207, Ala199, Leu390, Leu391	− 5.7	Ile523, Ala527, Ser530	Phe205, Val344, Tyr348, Val349, Leu352, Tyr385
Celecoxib	− 8.1	Gln192, Tyr355	Gly354, Lys358, Ile564, Phe580	− 8.0	Gln192, Gln350, Arg581	His90

Previous reports indicated that some of the isolated compounds have cytotoxic activity against melanoma cell lines, azelaic acid was reported to be active against murine B16 and human SK23 and HMB2 melanoma cell lines^49^, while 4-hydroxybenzoic acid showed cytotoxic activity aganist human SK-MEL-28 and murine B16-F0 cell lines^21^. Fusaric acid inhibited the melanoma tyrosinase as previously reported^22^. Further previous data concluded that the anticancer activity of azelaic acid could be attributed to its interference with synthesis of cellular DNA and/or oxidative phosphorylation, instead of tyrosinase inhibitory activity^25^. Another study on hydroxytyrosol indicated its effectiveness against A375, HT-144, and M74 melanoma cells^50^.

Since molecular docking is a beneficial approach for in silico determination of mechanism of action and biomolecular target, the current effort included docking studies to evaluate the potential binding of the isolated metabolites against a set of enzymes related to melanoma to discern which of them may be targeted by the metabolites. A total of four enzymes namely, quinone reductase 2 (QR2) (PDB ID: 1SG0)^51^, heat shock protein 90 (Hsp90) (PDB ID: 7K9S)^52^, B-Raf kinase (PDB ID: 3OG7)^53^, and pirin (PDB ID: 3ACL)^26^were used in the study. These enzymes are key players in melanoma biology; their inhibition has been reported to potentially affect melanoma either due to increased oxidative stress or induction of apoptosis or other various reported mechanisms^54–56^. All the studied compounds exhibited weak binding affinities to the investigated enzymes except for pirin where docking studies revealed that azelaic acid (**4**) and fusaric acid (**6**) exhibited various types of interactions with amino acids in its active site. Previous reports on pirin stated that its inhibition suppressed the migration of melanoma cells^26^.

Human pirin crystal structure studies revealed that it consists of two domains facing each other, they are similar in structure, folding and cavities; the N-terminal domain (amino acids 3–134) and the C-terminal domain (amino acids 143–290) and they are joined via a small linker (amino acids 134–143), a single iron metal ion lies in the N-terminal domain and it was reported to contribute to the stabilization of pirin crystal structure and to mediate its biological functions^26,57^.

**Figure 6:**
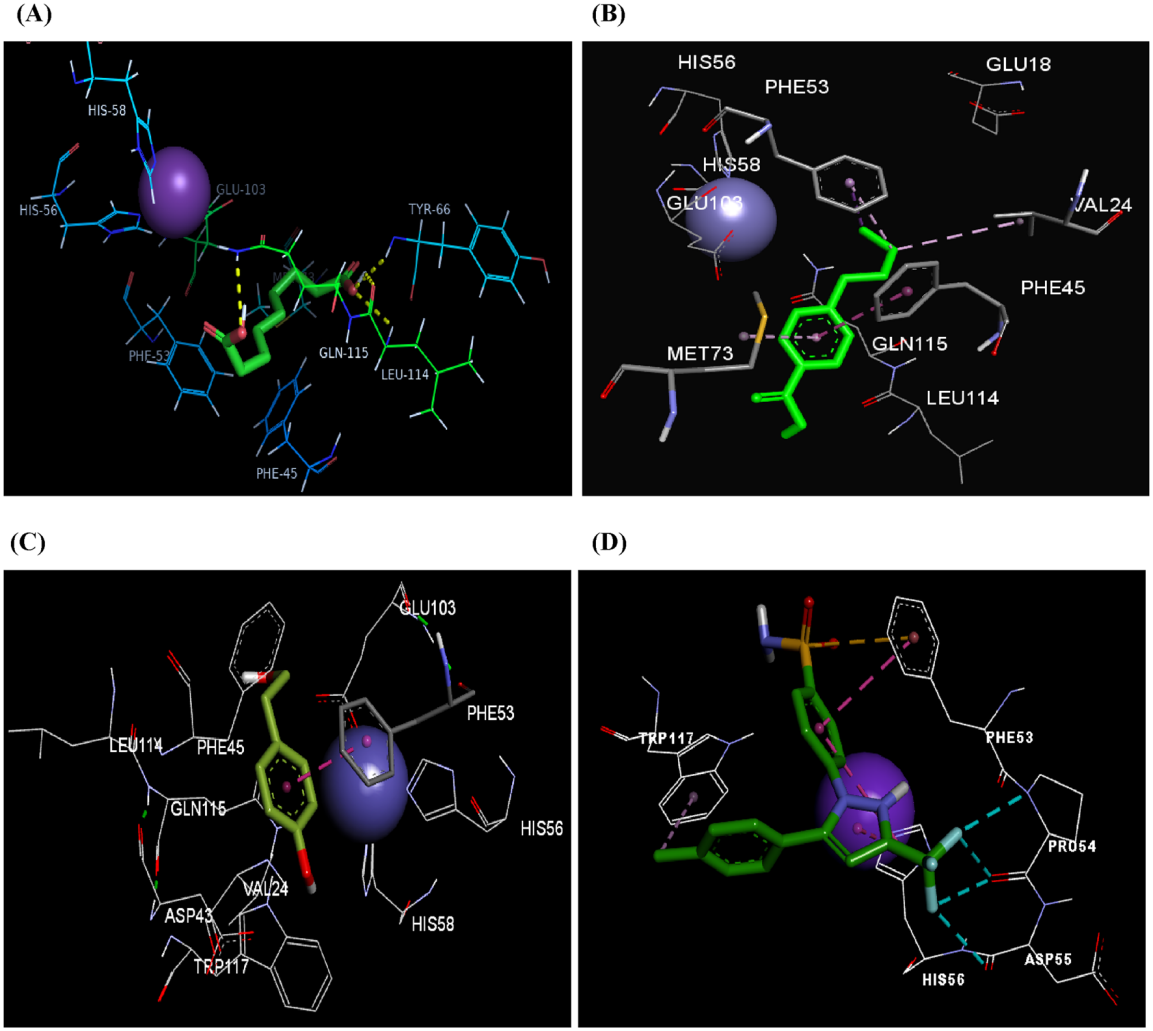
Binding modes of azelaic acid (**A**), fusaric acid (**B**), tyrosol (**C**), and celecoxib (**D**) into pirin binding site. Ligands are depicted in green tube models. Iron metal is shown as purple CPK representation. The amino acids shown in labeled line models. Hydrogen bonds are represented by yellow dashed lines, while hydrophobic interactions by pink and magenta dashed lines.

Analysis of the lowest energy docked pose of azelaic acid to pirin revealed two hydrogen bond interactions between one of its carboxylic groups and the amino acids Tyr66 and Leu114 while the other carboxylic group formed an additional hydrogen bonding with Gln115. Two π-alkyl interactions with the two amino acids; Phe45 and Phe53 have been revealed. Ten van der Waals contacts were observed with the pocket amino acids: Val24, Leu41, Asp43, Ser65, Tyr66, Leu67, Met85, Glu103, Gln115, and Trp117 (Fig. 6A). Fusaric acid binding to pirin exhibited various forms of hydrophobic interactions including π-π stacking with Phe45, alkyl and π-alkyl interactions with Met73, Val24, and Phe53, π-σ interaction with Phe53, and van der Waals interactions with Leu41, Asp43, Ser65, Tyr66, Leu67, Leu68, Met85, Asp89, Leu90, Gln91, Glu103, Gly113, Leu114, Gln115, and Trp117 (Fig. 6B), while tyrosol exhibited π-π stacking between its phenyl ring and the hydrophobic residue Phe53, in addition to twelve hydrophobic contacts with the amino acids Val24, Arg26, Leu41, Asp43, Phe45, Ser65, Tyr66, Met73, Glu103, Leu114, Gln115, and Trp117 (Fig. 6C).These results revealed that these compounds could incorporate themselves into the active site of pirin and bind tightly to its residues by various kinds of interactions suggesting that pirin may be a therapeutic target and a plausible mechanism for the anti-melanoma activity of these metabolites. Finally, the standards; celecoxib and kojic acid were docked into the binding site of pirin and they unveiled blinding scores of − 8.1 and − 5.7 kcal/mol; respectively, where celecoxib bound to pirin via halogen bonding of its fluorine atoms with Pro54 and Asp55 and stabilized by π-π stacking with His56, π-alkyl interactions to Trp117, and π-sulfur interactions between its sulfur and Phe53 (Fig. 6D). Celecoxib has been previously reported to inhibit cell growth in melanoma and reduce its invasiveness^58^, where some reports attributed this effect to its inhibition of COX-2 enzyme which is overexpressed in certain malignancies^59^ but this is still controversial since it demonstrated antitumor effect against low COX-2 expressing melanoma cell lines as well^60^. Interestingly, our results may add a new guidance to understand the mechanisms of celecoxib action against melanoma cells and initiate a new avenue for future research.

Based on docking data, our work proposed pirin as a new molecular mechanism underlying azelaic acid and fusaric acid anti-melanoma activity. Further in vitro and in vivo studies are suggested to validate these findings with possible structure modifications to enhance the activity.

## Conclusion

In conclusion, 4-hydroxybenzaldehyde (**1**), 4-hydroxybenzoic acid (**2**), tyrosol (**3**), azelaic acid (**4**), malic acid (**5**) and fusaric acid (**6**) were isolated from the endophytic fungus *Fusarium solani* cultured from the root of *Euphorbia tirucalli* plant. The anti-inflammatory activity studies revealed that azelaic acid exhibited potent and selective COX-2 inhibitory activity. Also, among the isolated compounds, azelaic acid revealed the highest anti-tyrosinase activity. In silico studies into cyclooxygenases were in good accord with in vitro results and thus revealing additional mechanism by which azelaic acid exerts its anti-inflammatory activity. Molecular docking studies against a set of enzymes related to melanoma revealed high binding affinities of azelaic acid and fusaric acid in addition to the standard celecoxib to the active site of pirin that could be a plausible target for their anti-melanoma activities. Meanwhile, confirming these findings requires further future studies.

## Experimental section

General instruments and chemicals are provided in the Supplementary material.

### Plant material

*Euphorbia tirucalli* plant was gathered in March 2018 from Cactus Farm in Tahanop, Shebin El-Qanater, Qalubiya, Egypt. The plant under investigation was authenticated by Dr. Abdelhalim Mohamed (Phyto-taxonomy Department, Agricultural Research Institute, Giza, Egypt), and a voucher sample (BUPD-61) was placed in the Pharmacognosy Department Herbarium, Faculty of Pharmacy, Beni-Suef University.

### Isolation of the endophytic fungus from *Euphorbiatirucalli*

*E. tirucalli* was collected and uprooted. The plant roots were rinsed in streaming tap water to clear out the dust particles, soil sand, and other external microorganisms^61^. The separated plant roots were cut into 2 mm segments and the surface was sterilized for 1 min by submerging in ethyl alcohol 70% then for 2 min in 5% sodium hypochlorite and finally washed with sterilized demineralized water for 1 min. To remove the moisture, the explant parts were pressed in the sterilized tissue paper. The separated explant parts were transferred and cultivated on the Petri dishes containing SDA (Sabouraud Dextrose Agar) provided with 150 mg/L chloramphenicol. The Petri dishes containing the explants were incubated at 27 °C for 14 days and fungus growth was daily monitored. New petri dishes with SDA devoid of antibiotics were used to carefully transfer the developing fungus from the explant samples^62^.

### Morphological features of the recovered isolate

Successive subcultures on SDA (Sabouraud dextrose agar) were done to purify pure fungal isolate. The fungal colony was grown on SDA for 10 days, then the cultural appearances (colony color and pigmentations) were observed. For microscopic analysis of microconidia and conidiospore staining of fungal component was done with lactophenol cotton blue stain.

### Molecular identification of the endophytic fungus

Hundred milligrams of fungal mycelium was transferred to a sterile mortar previously cooled with liquid nitrogen. The mycelium was solidified by addition of 1 ml of liquid nitrogen. Using a sterile pestle, the mycelium was finely ground for 10 min. The ground mycelium was recovered using 2 ml of lysis solution (20 mM EDTA, 10 mM Tris (pH 8.0), 1% Triton X100 (v/v)) then incubated at 37 °C for 60 min. Subsequently, 2 ml of 5 M NaCl and 1 ml of 1% (w/v) cetyltrimethylammonium bromide were added into the previous mixture and incubated for 45 min. at 65 °C. The resultant mixture was centrifuged at 8000 rpm for 10 min, the deposit was discarded, and the supernatant was subjected to further purification. The DNA was purified and concentrated using phenol/chloroform method. The method was modified from previously published method^63^. PCR and sequence analysis of ITS gene was done at Macrogen (Macrogen, South Korea). DNA sequence was aligned using blast database^64^ and the retrieved related sequences was aligned, and phylogenetic tree was constructed with MEGA 11^65^.

### Cultivation and secondary metabolites extraction

#### Large scale fermentation

Forty Erlenmeyer flasks (250 ml) containing 100 ml (SDB) were used for inoculation of the isolated fungus after being autoclaved for 15 min. at 121 °C. Static condition for 10 days at 28 °C was applied for fungal growth. After 10 days, 40 L of SDB were prepared and autoclaved for 15 min. at 121 °C for scaling up. The formerly prepared 100 ml broth cultures (40 flasks) were transmitted to the 40 L liquid broth under laminar flow. Lastly, for 10 days, the large-scale culture broth was kept at room temperature (about 30 °C).

### Preparation of the endophytic fungus extract

The culture was filtered under laminar flow using filter paper Whatman No.1 and divided into mycelium and filtrate. Successive extraction of the filtrate was carried out using equal volumes of EtOAc (Ethyl acetate) three times, then, the EtOAc layers were combined, while the frozen mycelia were crushed and extracted by equal volumes of EtOAc three times with aid of ultrasonic treatment. The collected EtOAc extract was evaporated under reduced pressure till dryness to afford a crude broth extract (2.8 g).

### Exploration of the ethyl acetate extract of endophytic fungus

The crude EtOAc extract (2.8 g) was subjected to column chromatography using a silica gel stationary phase (85 g, 160 × 2.5 cm), and mobile phase *n*-hexane and gradually increased polarity using ethyl acetate in 5% increments until 100%, then the polarity increased gradually with methanol in 10% increments until 100%. Five main fractions (**A**–**E**) were combined and evaporated separately under reduced pressure. Further chromatographic techniques were used for isolation of six metabolites: compound **C**_**1**_ (40 mg) as a buff powder, while compounds **C**_**2**_ (35 mg), **C**_**3**_ (50 mg), **C**_**4**_ (45 mg), **C**_**5**_ (38 mg) and **C**_**6**_ (30 mg) as white powder. The full method is provided in the Supplementary materials.

### Biological investigation

#### COX-1 and COX-2 inhibition assay

The anti-inflammatory capacities of the *F. solani* extract together with the six isolated metabolites were investigated via evaluating their inhibitory activities against both isoforms of the cyclooxygenase enzyme COX-1 and COX-2. The full method is provided in the Supplementary materials.

### Tyrosinase inhibitory assay

The anti-tyrosinase capacities of the *F. solani* extract together with the six isolated metabolites were investigated via evaluating their inhibitory activities against tyrosinase enzyme. The full method is provided in the Supplementary materials.

### Molecular docking

All used ligands structures were built using Chem & Bio 3D 12.0 software where their energy was minimized using the MM2 force field and then converted to PDB format. The X-ray crystallographic structures of human cyclooxygenase 1 (PDB ID: 6Y3C)^66^, human cyclooxygenase 2 (PDB ID: 5IKV)^44^, quinone reductase 2 (PDB ID: 1SG0)^51^ heat shock protein 90 (PDB ID: 7K9S)^52^, B-Raf kinase (PDB ID: 3OG7)^53^, and pirin (PDB ID: 3ACL)^26^ were obtained from the Protein Data Bank (https://www.rcsb.org/structure). Protein preparation step for each receptor has been performed. During protein preparation, water molecules, hetatoms, and ligand were removed, missing polar hydrogen atoms were added, and Kollman charges were added. Molecular docking studies were performed using Pyrx, AutoDock vina software after locating the Grid box to define the binding site. XYZ coordinates were set as; 6Y3C: − 33.24, − 43.08, 8.34; 5IKV: 167.78, 184.53, 188.99; 1SG0: 30.08, 14.42, 16.21; 7K9S: − 12.66, − 8.18, − 33.20; 3OG7: − 2.35, − 3.03, − 18.92; 3ACL: − 19.64, 13.52, − 13.08. The docked compounds binding interactions with each receptor were visualized and investigated using Pymol molecular graphics system version 2.5.5, Discovery studio visualizer v21.1.0.20298 and the web tool: “Protein–Ligand Interaction Profiler” at https://plip-tool.biotec.tu-dresden.de/plip-web/plip/.

### Statistical analysis

The experiments in this study were conducted in triplicate, and the data were presented as means ± standard errors of the means (SEM). Application of the Graph Pad Prism 6 program (San Diego, CA, USA) was used to do the analysis. *P* values ˂ 0.05 were regarded as statistically significant relative to the standard medication in each assay. An ANOVA test was performed to compare the data to the standard drugs, followed by a Tukey–Kramer post ANOVA test.

### Ethics approval and consent to participate

*Euphorbia tirucalli* aerial parts were collected at Cactus Farm, Tahanop, Shebin El-Qanater, Qalubiya, Egypt in October 2014. The collection of *E. tirucalli* root and experimental work complied with relevant institutional, national, and international guidelines and legislation. Samples of the collected plant were introduced to Phyto-taxonomy Department, Agricultural Research Institute, Cairo, Egypt where it has been identified by Dr. Abdelhalim Mohamed, and a voucher specimen labeled as (BUPD-61) has been deposited in Herbarium of Pharmacognosy Department, Faculty of Pharmacy, Beni-Suef University, Beni-Suef, Egypt. The collection of *E. tirucalli* does not require individual permission.

### Supplementary Information

Supplementary Information.

## Data Availability

The corresponding author can provide the data supporting the study's findings upon reasonable request.
