# Supplementary material for: New insights into the anti-inflammatory and anti-melanoma mechanisms of action of azelaic acid and other Fusarium solani metabolites via in vitro and in silico studies
Source: Sci Rep. 2024 Jun 22;14:14370. doi: 10.1038/s41598-024-63958-0 (PMC11193793; doi:10.1038/s41598-024-63958-0)
Supplement: Supplementary file 1 — Supplementary Information. [file 41598_2024_63958_MOESM1_ESM.pdf]

New Insights into the Anti-inflammatory and Anti-melanoma Mechanisms of Action of Azelaic acid and other *Fusarium solani* metabolites *via in vitro* and *in silico* studies

Mona Ismail[1†], Mohamed A. Zaki[1†], Marwa H. A. Hassan[1], Enas I. A. Mohamed[1], Ahmed F. Azmy[2], Abeer Moawad[1]*, Rabab Mohammed[1]*

[1]Department of Pharmacognosy, Faculty of Pharmacy, Beni-Suef University, Beni-Suef 62514, Egypt.
[2]Department of Microbiology and Immunology, Faculty of Pharmacy, Beni-Suef University, Beni-Suef 62514, Egypt

[†]These authors contributed equally to this work.

Authors

Mona Ismail: monaismail92@gmail.com, mona.ismail@pharm.bsu.edu.eg, 0000-0002-9663-1274
Mohamed A. Zaki: mohamed.zaki@pharm.bsu.edu.eg, 0000-0003-4889-8836
Marwa H. A. Hassan: mh_elsefy@yahoo.com, marwa.hassan@pharm.bsu.edu.eg, 0000-0002-0952-5984
Enas I. A. Mohamed: enas.mohamed@pharm.bsu.edu.eg, 0000-0002-6943-9383
Ahmed F. Azmy: ahmed.abdelaziz@pharm.bsu.edu.eg, 0000-0002-7347-9564
Abeer Sayed: abeermoawad77@gmail.com, abeer.moawad@pharm.bsu.edu.eg, 0000-0002-0228-6757
Rabab Mohammed: rababmohammed@pharm.bsu.edu.eg, rmwork06@yahoo.com, 0000-0001-9683-4250

***Correspondence:**
Prof. Dr. Rabab Mohammed, rababmohammed@pharm.bsu.edu.eg, rmwork06@yahoo.com.

Mobile No.: +201202442204

Prof. Dr. Abeer Sayed, abeermoawad77@gmail.com, abeer.moawad@pharm.bsu.edu.eg.

Mobile No.: +201148895396

Supplementary material:

Table of content:

	Content	Page No.
1.	**Experimental section**	3
2.	**Spectral data of isolated compounds**	6
3.	**List of Figures:**	7
4.	**Figure S_1:** ^{1}H NMR spectrum of compound C_1 4-hydroxy benzaldehyde (400 MHz, CD_3OD)	9
5.	**Figure S_2:** DEPT-Q spectrum of compound C_1 4-hydroxy benzaldehyde (100 MHz, CD_3OD)	10
6.	**Figure S_3:** ^{1}H NMR spectrum of compound C_2 4-hydroxy benzoic acid (400 MHz, CD_3OD)	11
7.	**Figure S_4:** DEPT-Q spectrum of compound C_2 4-hydroxy benzoic acid (100 MHz, CD_3OD)	12
8.	**Figure S_5:** ^{1} H NMR spectrum of compound C_3 Tyrosol (400 MHz, CD_3OD)	13
9.	**Figure S_6:** DEPT-Q spectrum of compound C_3 Tyrosol (100 MHz, CD_3OD)	14
10.	**Figure S_7:** ^{1}H NMR spectrum of compound C_4 Azelaic acid (400 MHz, CD_3OD)	15
11.	**Figure S_8:** DEPT-Q spectrum of compound C_4 Azelaic acid (100 MHz, CD_3OD)	16
12.	**Figure S_9:** ^{1}H NMR spectrum of compound C_5 Malic acid (400 MHz, CD_3OD)	17
13.	**Figure S_{10}:** DEPT-Q spectrum of compound C_5 Malic acid (100 MHz, CD_3OD)	18
14.	**Figure S_{11}:** ^{1}H NMR spectrum of compound C_6 Fusaric acid (400 MHz, CD_3OD)	19
15.	**Figure S_{12}:** DEPT-Q spectrum of compound C_6 Fusaric acid (100 MHz, CD_3OD)	20
16.	**Table S1:** Docking scores of isolated compounds from the endophytic fungus Fusarium solani against the enzymes QR2, Hsp90, B-Raf kinase, and pirin	21
17.	**References**	22

Experimental section

ITS sequence

**AACGTTGCCTCGGCGGGAACAGACGGCCCCGTAACACGGGCCGCCCCCGCCAGAGGACCCCCTAAC
TCTGTTTCTATAATGTTTCTTCTGAGTAAACAAGCAAATAAATTAAAACTTTCAACAACGGATCTCTT
GGCTCTGGCATCGATGAAGAACGCAGCGAAATGCGATAAGTAATGTGAATTGCAGAATTCAGTGAAT
CATCGAATCTTTGAACGCACATTGCGCCCGCCAGTATTCTGGCGGGCATGCCTGTTCGAGCGTCATT
ACAACCCTCAGGCCCCCGGGCCTGGCGTTGGGGATCGGCGGAAGCCCCCTGCGGGCACAACGCCGT
CCCCCAAATACAGTGGCGGTCCCGCCGCAGCTTCCATTGCGTAGTAGCTAACACCTCGCAACTGGAG
AGCGGCGCGGCCACGCCGTAAAACACCCAACTTCTGAATGTTGACCTCGAATCAGGTAGGAATACCC
GCCGAGTTATACAACTCATCAACCCTGTGAACATACCTATAACGTTGCCTCGGCGGGAACAGACGGC
CCCGTAACACGGGCCGCCCCCGCCAGAGGACCCCCTAACTCTGTTTCTATAATGTTTCTTCTGAGTA
AACAAGCAAATAAATTAAAACTTTCAACAACGGATCTCTTGGCTCTGGCATCGATGAAGAACGCAGC
GAAATGCGATAAGTAATGTGAATTGCAGAATTCAGTGAATCATCGAATCTTTGAACGCACATTGCGC
CCGCCAGTATTCTGGCGGGCATGCCTGTTCGAGCGTCATTACAACCCTCAGGCCCCCGGGCCTGGC
GTTGGGGATCGGCGGAAGCCCCCTGCGGGCACAACGCCGTCCCCCAAATACAGTGGCGGTCCCGCC
GCAGCTTCCATTGCGTAGTAGCTAACACCTCGCAACTGGAGAGCGGCGCGGCCACGCCGTAA**

General instruments and chemicals

Brucker NMR spectrometer was used to acquire NMR data running at 400, and 100 MHz for ^{1}H and ^{13}C and DEPTQ, respectively. For chromatographic separation, silica gel 60 (Sigma-Aldrich Chemicals, Darmstadt, Germany), and sephadex LH-20 (0.25-0.1 mm, GE Healthcare Bio-Sciences AB SE 75184 Uppsala, Sweden), were used for column chromatography (CC). TLC plates (Fluka precoated silica gel F_{254}) were used for column monitoring. Multi-well spectrophotometer (fluorescence plate reader) was used for *in vitro* activities. Solvents used for NMR analysis were MeOH-*d*$_4$ (CD_3OD), dimethyl sulphoxide-*d*$_6$ (DMSO), and chloroform-*d*$_1$ ($CDCl_3$) were purchased from (Sigma-Aldrich, Germany). L-dihydroxyphenylalanine (L-DOPA), celecoxib, DMSO, and indomethacin were purchased from Merck (Darmstadt, Germany) for *in vitro* activities. Other Solvents like *n*-hexane, ethyl acetate (EtOAc), and methanol (MeOH) were obtained from El-Nasr Company for Pharmaceuticals and Chemicals, Egypt.

Exploration of the ethyl acetate extract of endophytic fungus

The crude EtOAc extract (2.8 g) was subjected to column chromatography using a silica gel stationary phase (85 g, 160 × 2.5 cm), and mobile phase *n*-hexane and gradually increased polarity using ethyl acetate in 5% increments until 100%, then the polarity increased gradually with methanol in 10% increments until 100%. Thin layer chromatography (TLC) and the universal spray reagent *p*-anisaldehyde were used for monitoring the collected fractions similarity. Forty-five fractions (50

ml, each) were collected, and similar fractions were combined together and evaporated separately under reduced pressure till dryness to yield five major fractions (A-E). **Fraction A** (9-12, 0.2 g, eluted with 90% - 10% *n*-hexane in EtOAc) was chromatographed a second time using a silica gel column (10 g, 20 × 1.5 cm) and separated with *n*-hexane-EtOAc of 2.5% increasing polarity. Thiry sub-fractions (25 ml, each) were collected, and similar fractions were collected and evaporated to dryness yielding sub-fraction A_1. Sub-fraction A_1 (21-24, 0.1 g, eluted with 95%-5% *n*-hexane in EtOAc) was found to contain one major spot and was subjected to further purification using a column of Sephadex LH-20 (15g, 50 × 1.2 cm) and MeOH (100%) used as eluent to yield compound $\mathbf{C_1}$ (40 mg) as a buff powder. **Fraction B** (13-16, 0.3 g, eluted with 85% - 15% *n*-hexane in EtOAc) was chromatographed using column of silica gel (15 g, 25 × 2 cm) and *n*-hexane eluent with 2.5% increments of EtOAc. Thirty-five fractions (25 ml, each) were collected, and similar sub-fractions gathered and evaporated to a dry state to give sub-fraction $B_{1.}$ Sub-fraction B_1 (25-28, 0.15 g, eluted with 80%-20% *n*-hexane in EtOAc) was further refined over a column of Sephadex LH-20 (15g, 50 × 1.2 cm) and eluted with MeOH (100%) to afford compound $\mathbf{C_2}$ (35 mg) and $\mathbf{C_3}$ (50 mg) as white powder. **Fraction C** (19-22, 0.2 g, eluted with 80% - 20% *n*-hexane in EtOAc) was chromatographed using a column of silica gel (10 g, 20 × 1.5 cm) and separated with *n*-hexane and EtOAc with 2.5% of increasing polarity. Thirty sub-fractions similar sub-fractions (20 ml, each) were gathered and evaporated separately to a dry state to give sub-fraction C_1 (19-20, 0.1 g, eluted with77.5%- 22.5% *n*-hexane in EtOAc), was further purified using a column of Sephadex LH-20 (15 g, 50 x 1.2 cm) and eluted using MeOH (100%) to give compound $\mathbf{C_4}$ (45 mg) as white powder. **Fraction D** (25-30, 0.2 g, eluted with 75% - 25% *n*-hexane in EtOAc) was subjected to a column of a silica gel (10 g, 20 × 1.5 cm) and eluted with *n*-hexane and EtOAc of 2.5% increments in polarity. Thirty-five sub-fractions (20 ml, each) were collected and similar sub-fractions gathered and evaporated to a dry state to give sub-fraction D_1 (25-27, 0.11 g, eluted with 72.5% - 27.5% *n*-hexane in EtOAc) that was refined over Sephadex LH-20 column (15 g, 50 x 1.2 cm) and eluted with 100% methanol to yield compound $\mathbf{C_5}$ (38 mg) as white powder. Finally, **Fraction E** (31-37, 0.2 g, eluted with 70% - 30% *n*-hexane in EtOAc) was chromatographed on a column of silica gel (10 g, 20 × 1.5 cm) and solvent system *n*-hexane and EtOAc with 2.5% increments. Thirty-five sub-fractions were collected and similar sub-fractions gathered and evaporated to a dry state to give sub-fraction E_1 (25-30, 0.09 g, eluted with 75% - 25% *n*-hexane in EtOAc), which was further purified using a column of Sephadex LH-20 (15 g, 50 x 1.2 cm) and eluted with methanol (100%), affording compound $\mathbf{C_6}$ (30 mg) as white powder.

COX-1 and COX-2 inhibition assay

The anti-inflammatory capacities of the *F. solani* extract together with the six isolated metabolites were investigated via evaluating their inhibitory activities against both isoforms of the cyclooxygenase enzyme COX-1 and COX-2.

Serial dilutions (100, 10, 1, 0.1, and 0.01 μg/mL) of the *F. solani* extract and the isolated metabolites were prepared and solvated in DMSO, then estimated for their abilities to inhibit the synthesis of prostaglandin using the kits for screening test for COX inhibitors (BioVision, Inc., USA, Mountain View, Catalog # K548-100 and Catalog # K547-100, for COX-1 and COX-2, respectively); ovine COX-1 and recombinant human COX-2. The investigational methodology was carried out following the provider's recommendations. Arachidonic acid is being converted into prostaglandin (PGH2) by the cyclooxygenase enzyme (COX). During the reduction, the PGF2α formed in PGH2 via stannous chloride was evaluated via ELISA (enzyme-linked immunoassay). This bioassay is based on the competition among an antibody; PGspecific and a cholinesterase conjugate; PG-acetyl. The developed (antibody-PG) was combined with an acetylcholinesterase substrate included in Ellman's reagent. Each sample (20 µL) was used, (10 µL) heme, and (10 µL) enzyme, were combined to the reaction buffer solution (160 µL) formed of (2 mM phenol and 5 mM ethylenediamine tetra acetate (EDTA) in 0.1 M Tris-HCl, pH 8), followed by incubation of the mixture in a water bath at 37 °C for 10 min. Subsequently, starting the COX reaction was done by adding 10 µL of arachidonic acid, with a final concentration of 100 mM, to the reaction mixture. Later, the COX reactions were ended after 2 min by adding saturated stannous chloride (30 µL), then inoculate for 5 min at room temperature. Afterwards, the PGF2α level was measured using ELISA. Subsequent move to a 96-well plate, inoculate the samples at room temperature for 18 h, then wash the plate to eliminate any residual reagents, followed by the addition of Ellman's reagent (200 µL) which contains an acetylcholinesterase substrate, and incubation at room temperature for 60 to 90 min. A spectrophotometer with a microplate reader at 410 nm was used to measure the produced color at 410 nm. Finally, the IC_{50} values of COX-1 and COX-2 inhibition were assessed by comparing the incubations treated with the sample to the control incubations, Indomethacin®, and Celecoxib®, respectively [1,2]. The data mean values from three measurements were used to calculate IC_{50} values.

Tyrosinase inhibitory assay

The anti-tyrosinase capacities of the *F. solani* extract together with the six isolated metabolites were investigated *via* evaluating their inhibitory activities against tyrosinase enzyme using the kits for screening test of tyrosinase enzyme inhibition (BioVision, Inc., USA, Mountain View, Catalog # K575-100).

First, all samples were dissolved using DMSO (reference drug, EtOAc extract and the isolated compounds) preparing different concentrations. The assay of tyrosinase inhibition was done as previously reported [3]. The mixture of assay composed of test solution (1900 μL) in (0.1 M) buffer phosphate pH 6.8 and freshly prepared enzyme solution (100 μL) which is composed of 15 U/mL in (0.1 M) buffer phosphate pH 6.8. The 30 min. of room temperature after pre-incubation, the reaction was started by adding substrate solution (1000 μL) which composed of L-DOPA (1.5 Mm) in buffer phosphate (0.1 M) pH 6.8. After 7 minutes of room temperature incubation, the mixture of the assay was analyzed for absorbance at

510 nm. Kojic acid, a recognized tyrosinase inhibitor, was utilized as reference drug. The activity of tyrosinase inhibition represented as % of inhibition that determined as $(1 - C/D) \times 100$, where C (The activity of the enzyme activity) and D (The activity of the enzyme together with the tested samples). The mean values of the data from three measurements were used to calculate IC_{50} values.

- **Spectral data of isolated compounds**

4-hydroxybenzaldehyde (1): buff powder, ^{1}H NMR (400 MHz, MeOD) δ 7.66 (2H, d, *J* = 8 Hz, H-2, 6), 6.84 (2H, d, *J* = 8 Hz, H-3, 5), 9.64 (1H, s, H-7).^{13}C NMR (100 MHz, MeOD) δ 128.81 (C-1), 132.25 (C-2 & C-6), 115.62 (C-3 & C-5), 163.51 (C-4), 191.76 (C-7)[4].

4-hydroxybenzoic acid (**2**): buff powder, ^{1}H NMR (400 MHz, MeOD) δ 7.91 (2H, d, *J* = 8 Hz, H-2, 6), 6.85 (2H, d, *J* = 8 Hz, H-3, 5).^{13}C NMR (100 MHz, MeOD) δ 121.20 (C-1), 131.89 (C-2 & C-6), 114.88 (C-3 & C-5), 161.86 (C-4), 169.39 (C-7)[5].

Tyrosol (**3**): white powder, ^{1}H NMR (400 MHz, MeOD) δ 3.72 (2H, t, *J* = 8 Hz, H-1), δ 2.74 (2H, t, *J* = 8 Hz, H-2), δ 7.04 (2H, d, *J* = 8 Hz, H-2′, 6′), 6.77 (2H, d, *J* = 8 Hz, H-3′, 5′).^{13}C NMR (100 MHz, MeOD) δ 63.29 (C-1), 37.97 (C-2), 129.87 (C-1′), 129.73 (C-2′, 6'), 114.99 (C-3′, 5'), 155.13 (C-4′)[6].

Azelaic acid (**4**): white powder, ^{1}H NMR (400 MHz, MeOD) δ 2.30 (4H, t, H-2), δ 1.37-1.39 (4H, m, H-4), δ 1.37-1.39 (2H, m, H-5), δ 1.62 (4H, m, H-3).^{13}C NMR (100 MHz, MeOD) δ 176.36 (C-1), 33.54 (C-2), 28.67 (C-4), 28.67 (C-5), 24.63 (C-3)[7].

Malic acid (**5**): white powder, ^{1}H NMR (400 MHz, MeOD) δ 4.54 (1H, q, *J* = 4, *J* = 12 Hz, H-3), δ 2.85 (1H, m, H-4a) & δ 2.73 (1H, m, H-4b). ^{13}C NMR (100 MHz, MeOD) δ 175.44 (C-1), 173.25 (C-2), 67.04 (C-3), 38.53 (C-4)[8].

Fusaric acid (**6**): white powder, ^{1}H NMR (400 MHz, MeOD) δ 7.76 (1H, d, *J* = 6.4 Hz, H-3), δ 8.17 (1H, d, *J* = 6.4 Hz, H-4), δ 8.77 (1H, s, H-6), δ 13.294 (1H, s, H-7), δ 2.704 (2H, s, H-8), δ 1.605 (2H, H-9), δ 1.317 (2H, H-10), δ 0.872 (3H, t, *J* = 6.4 Hz, H-11). ^{13}C NMR (100 MHz, MeOD) δ 145.22 (C-2), 124.72 (C-3), 138.65 (C-4), 143.04 (C-5), 147.59 (C-6), 165.47 (C-7), 32.86 (C-8), 32.74 (C-9), 22.21 (C-10), 13.72 (C-11)[9].

List of figures

- **Figure S1:** ^{1}H NMR spectrum of compound C_1 4-hydroxy benzaldehyde (400 MHz, CD_3OD)
- **Figure S2:** DEPT-Q spectrum of compound C_1 4-hydroxy benzaldehyde (100 MHz, CD_3OD)
- **Figure S3:** ^{1}H NMR spectrum of compound C_2 4-hydroxy benzoic acid (400 MHz, CD_3OD)
- **Figure S4:** DEPT-Q spectrum of compound C_2 4-hydroxy benzoic acid (100 MHz, CD_3OD)
- **Figure S5:** ^{1}H NMR spectrum of compound C_3 Tyrosol (400 MHz, CD_3OD)
- **Figure S6:** DEPT-Q spectrum of compound C_3 Tyrosol (100 MHz, CD_3OD)
- **Figure S7:** ^{1}H NMR spectrum of compound C_4 Azelaic acid (400 MHz, CD_3OD)
- **Figure S8:** DEPT-Q spectrum of compound C_4 Azelaic acid (100 MHz, CD_3OD)
- **Figure S9:** ^{1}H NMR spectrum of compound C_5 Malic acid (400 MHz, CD_3OD)
- **Figure S10:** DEPT-Q spectrum of compound C_5 Malic acid (100 MHz, CD_3OD)
- **Figure S11:** ^{1}H NMR spectrum of compound C_6 Fusaric acid (400 MHz, CD_3OD)
- **Figure S12:** DEPT-Q spectrum of compound C_6 Fusaric acid (100 MHz, CD_3OD)

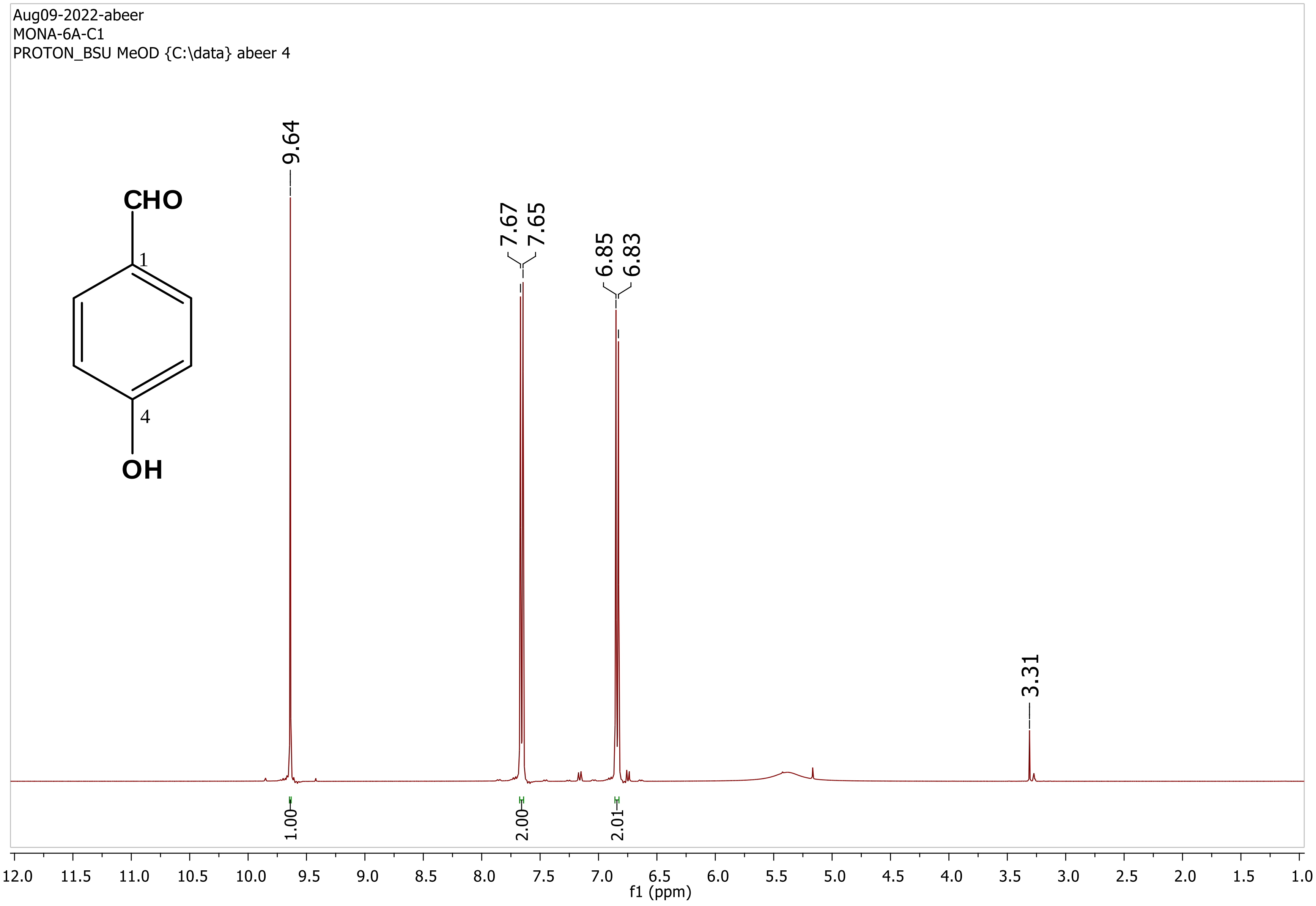

Figure S_1: ^1H NMR spectrum of compound C_1 <u>4-hydroxy benzaldehyde</u> (400 MHz, CD_3OD)

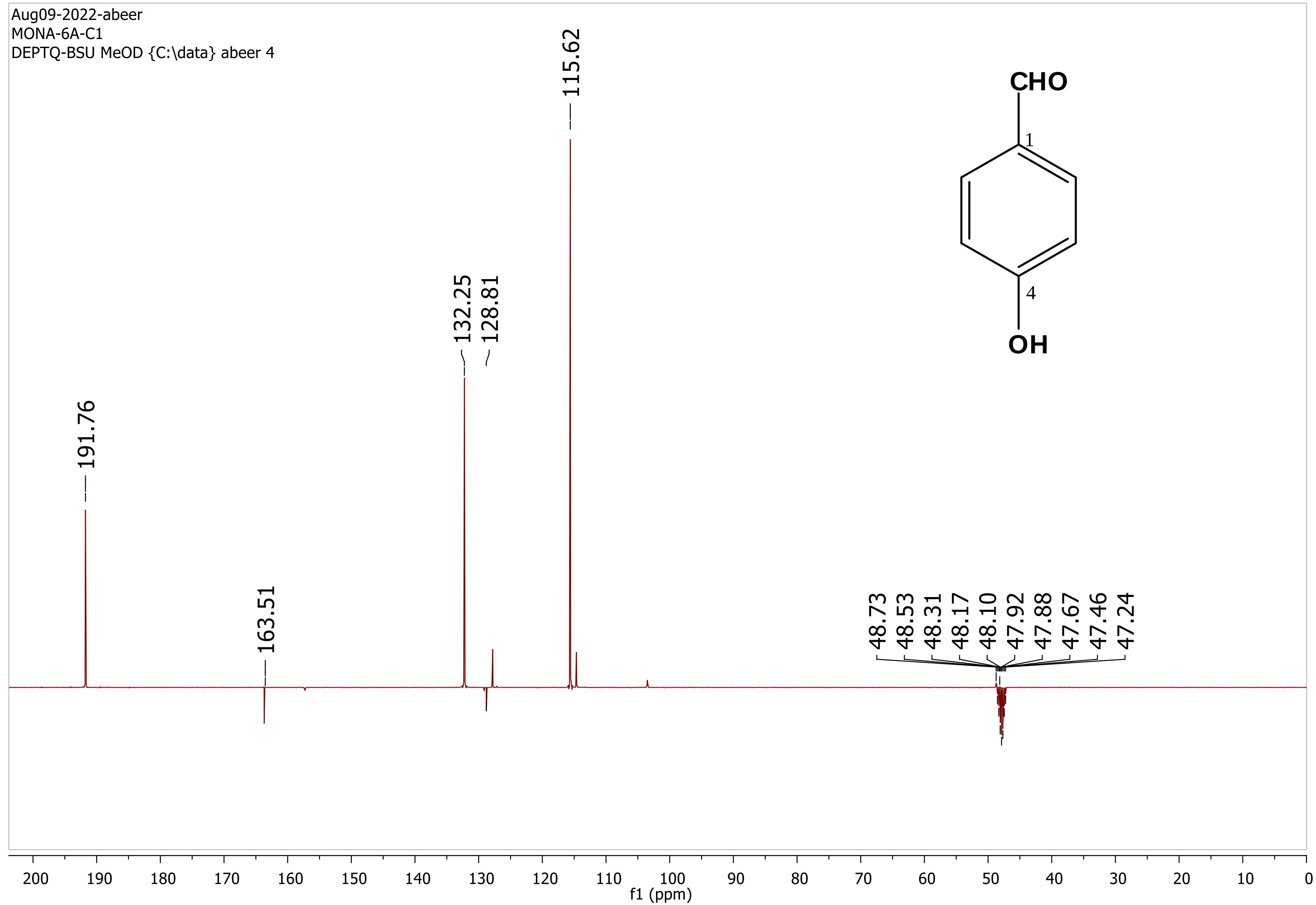

Figure S_2: DEPT-Q spectrum of compound C_1 4-hydroxy benzaldehyde (100 MHz, CD_3OD)

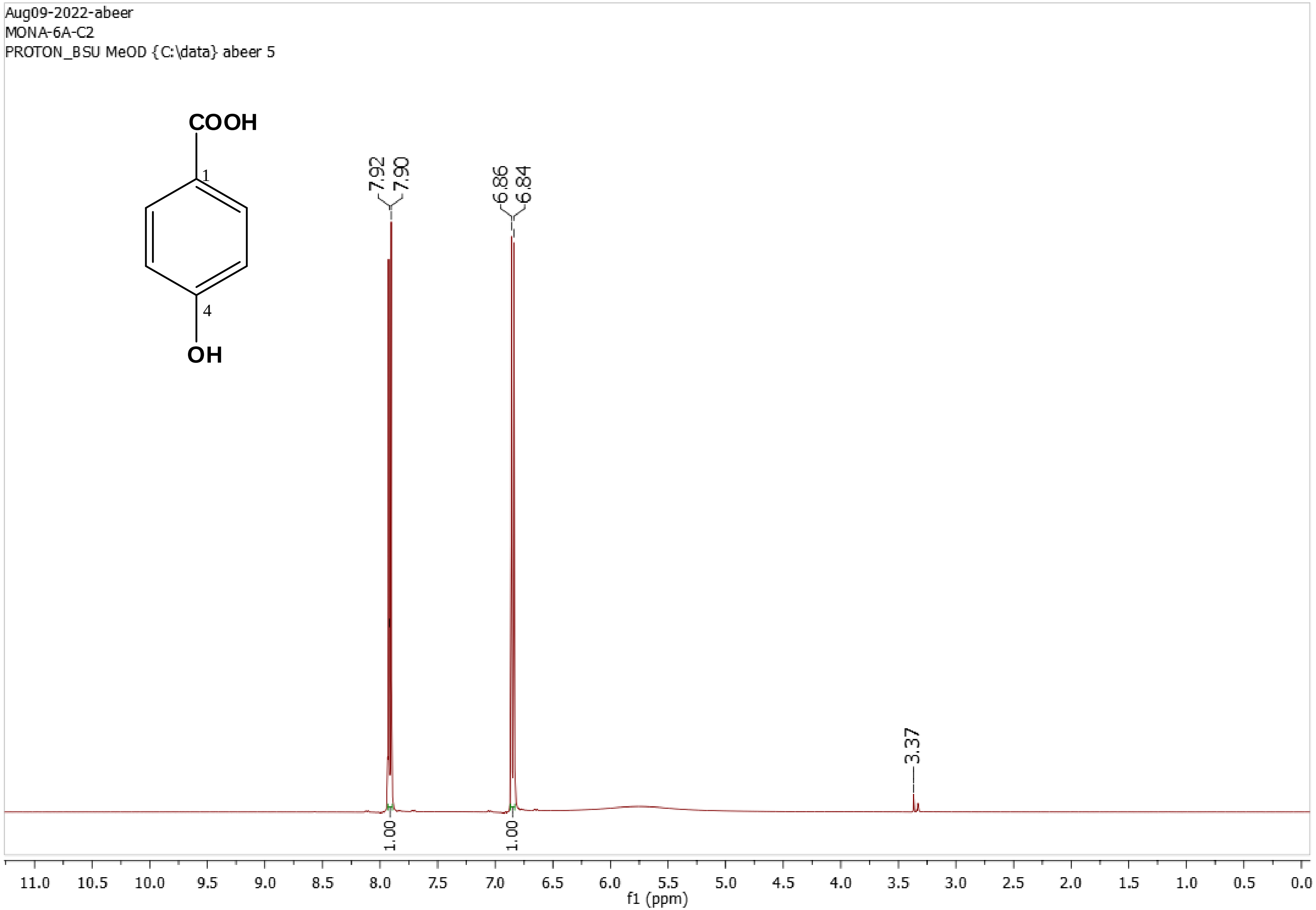

Figure S3: 1 H NMR spectrum of compound C2 <u>4-hydroxy benzoic acid</u> (400 MHz, CD_3OD)

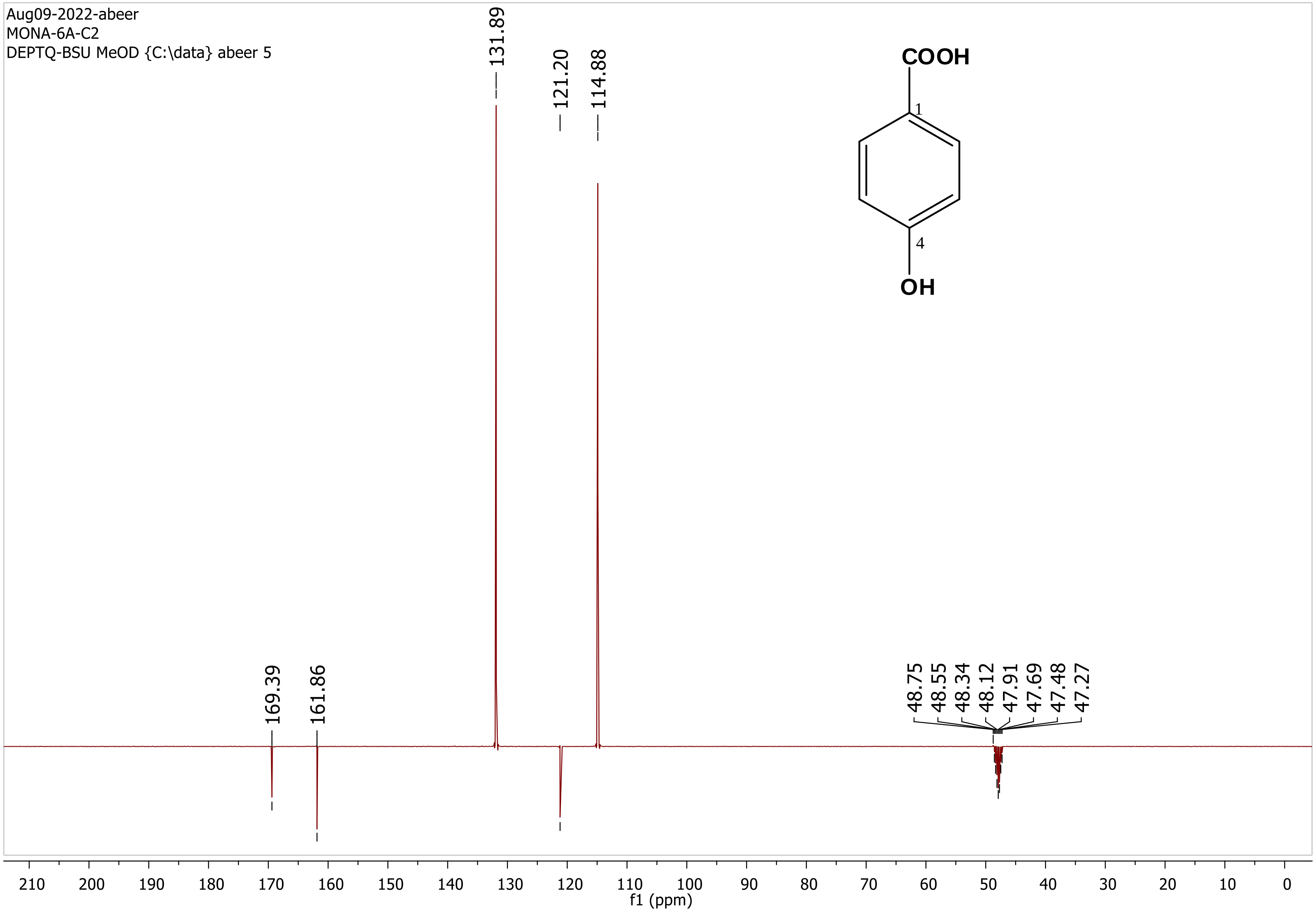

Figure S4: DEPT-Q spectrum of compound C2 4-hydroxy benzoic acid (100 MHz, CD_3OD)

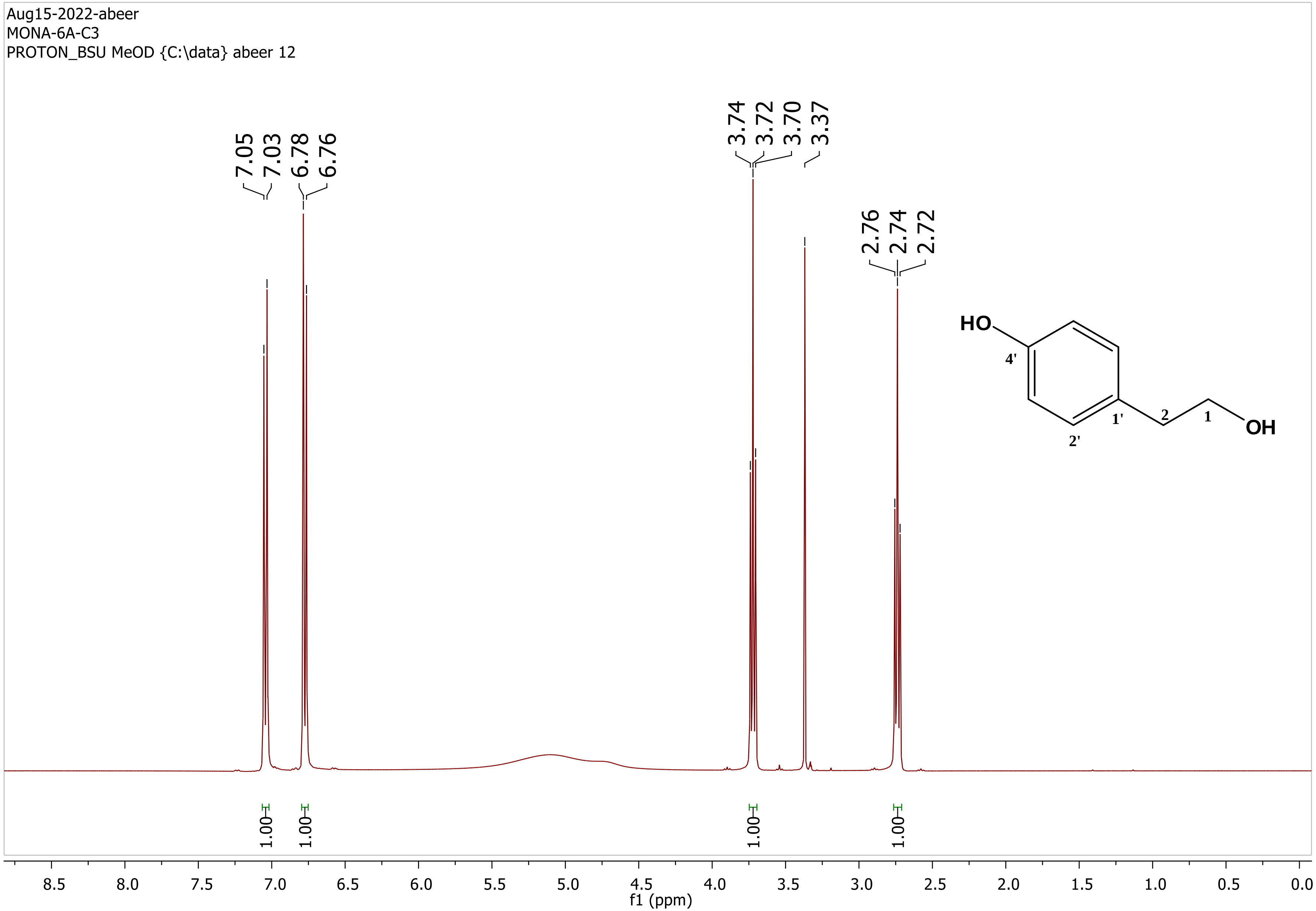

Figure S5: [1] H NMR spectrum of compound C_3 <u>Tyrosol</u> (400 MHz, CD_3OD)

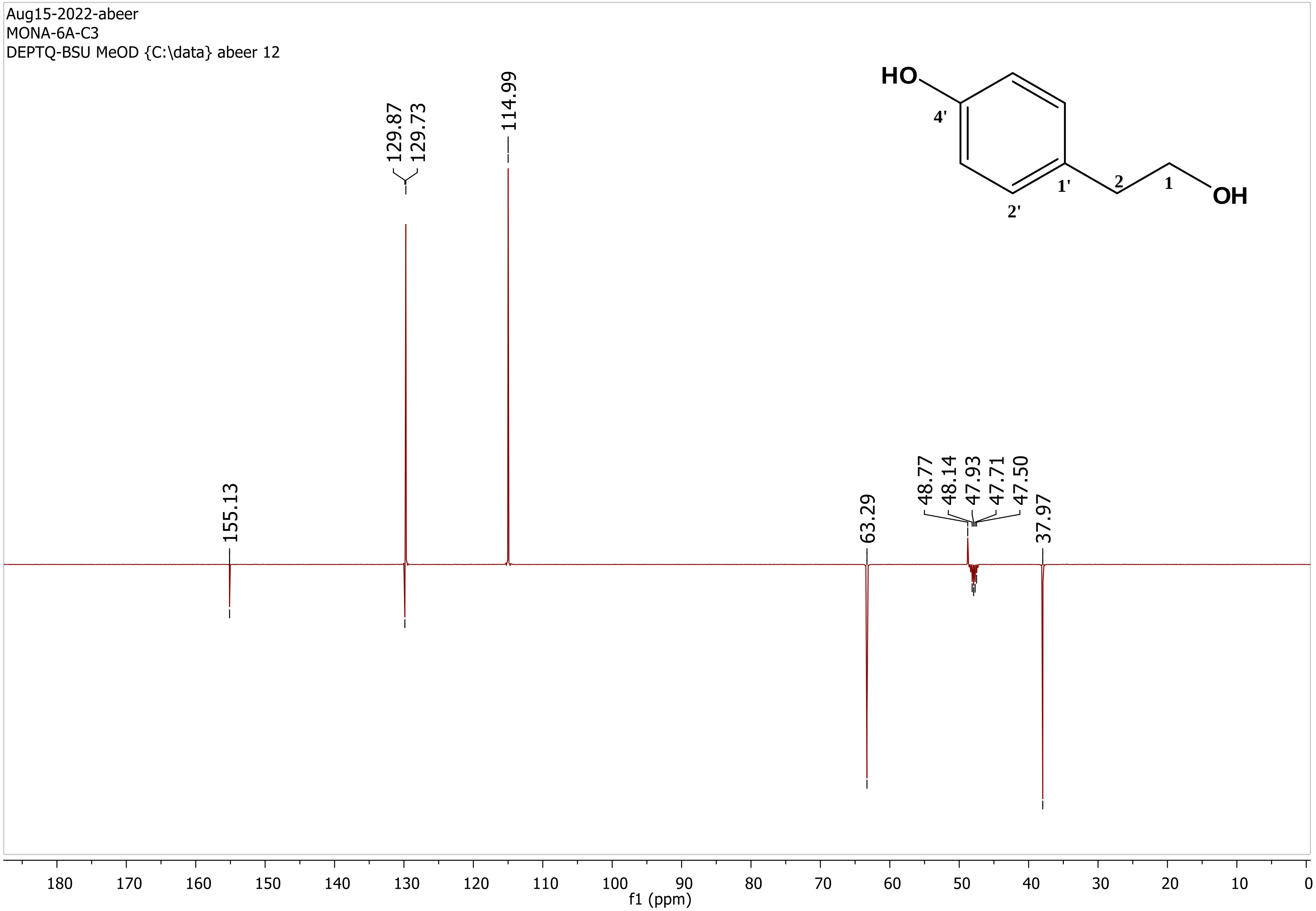

Figure S6: DEPT-Q spectrum of compound C3 Tyrosol (100 MHz, CD_3OD)

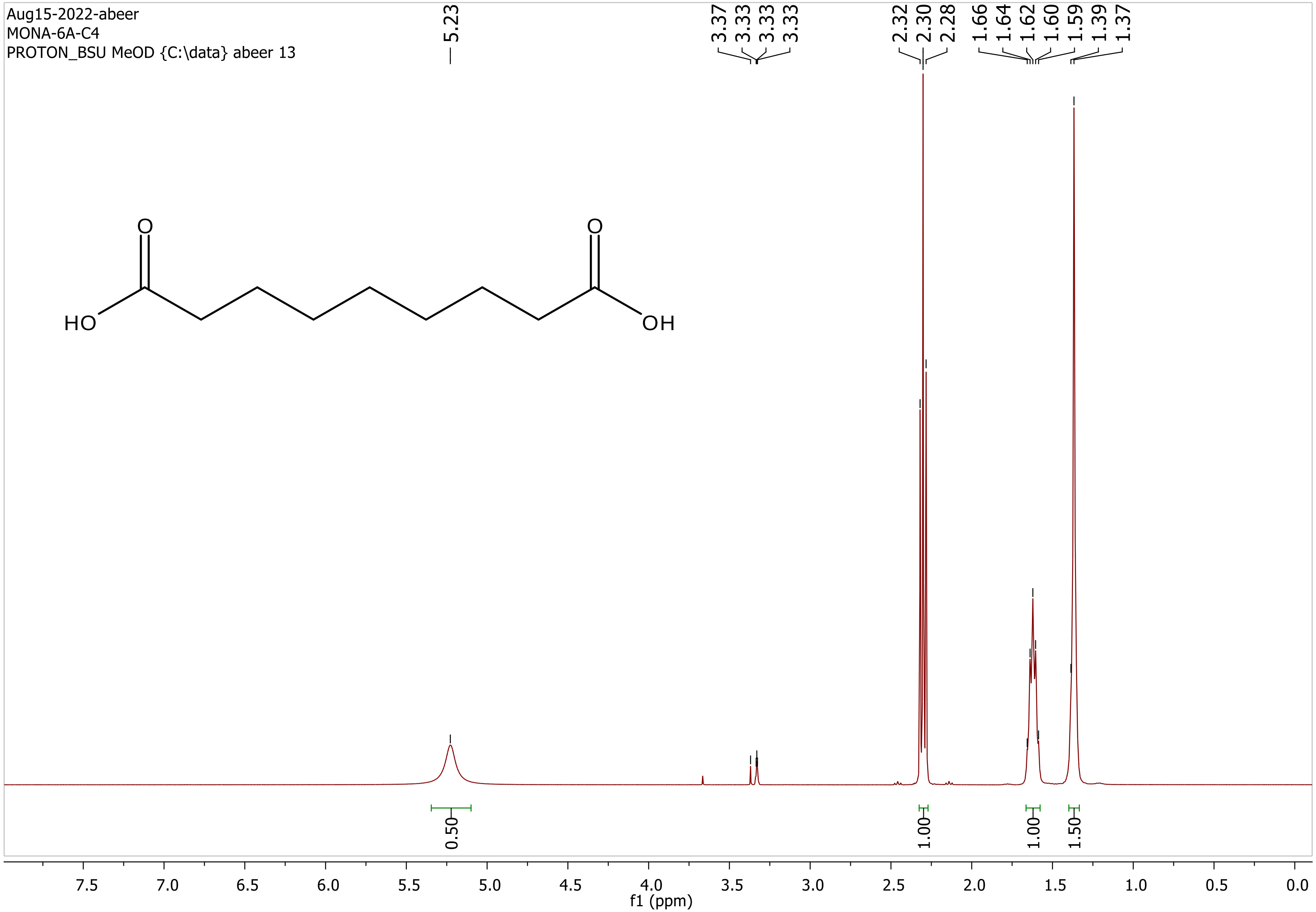

Figure S7: [1] H NMR spectrum of compound C_4 Azelaic acid (400 MHz, CD_3OD)

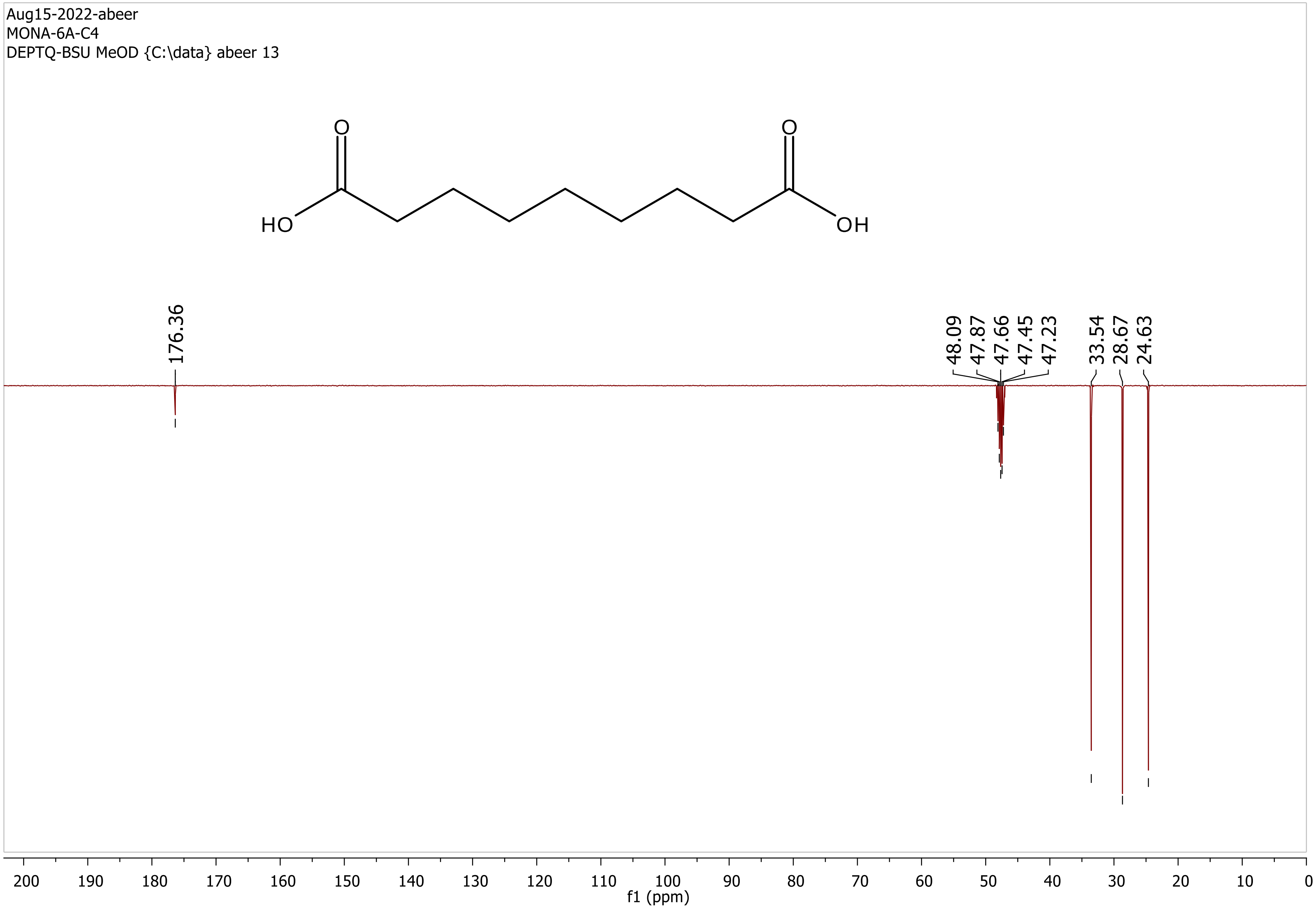

Figure S8: DEPT-Q spectrum of compound C4 Azelaic acid (100 MHz, CD_3OD)

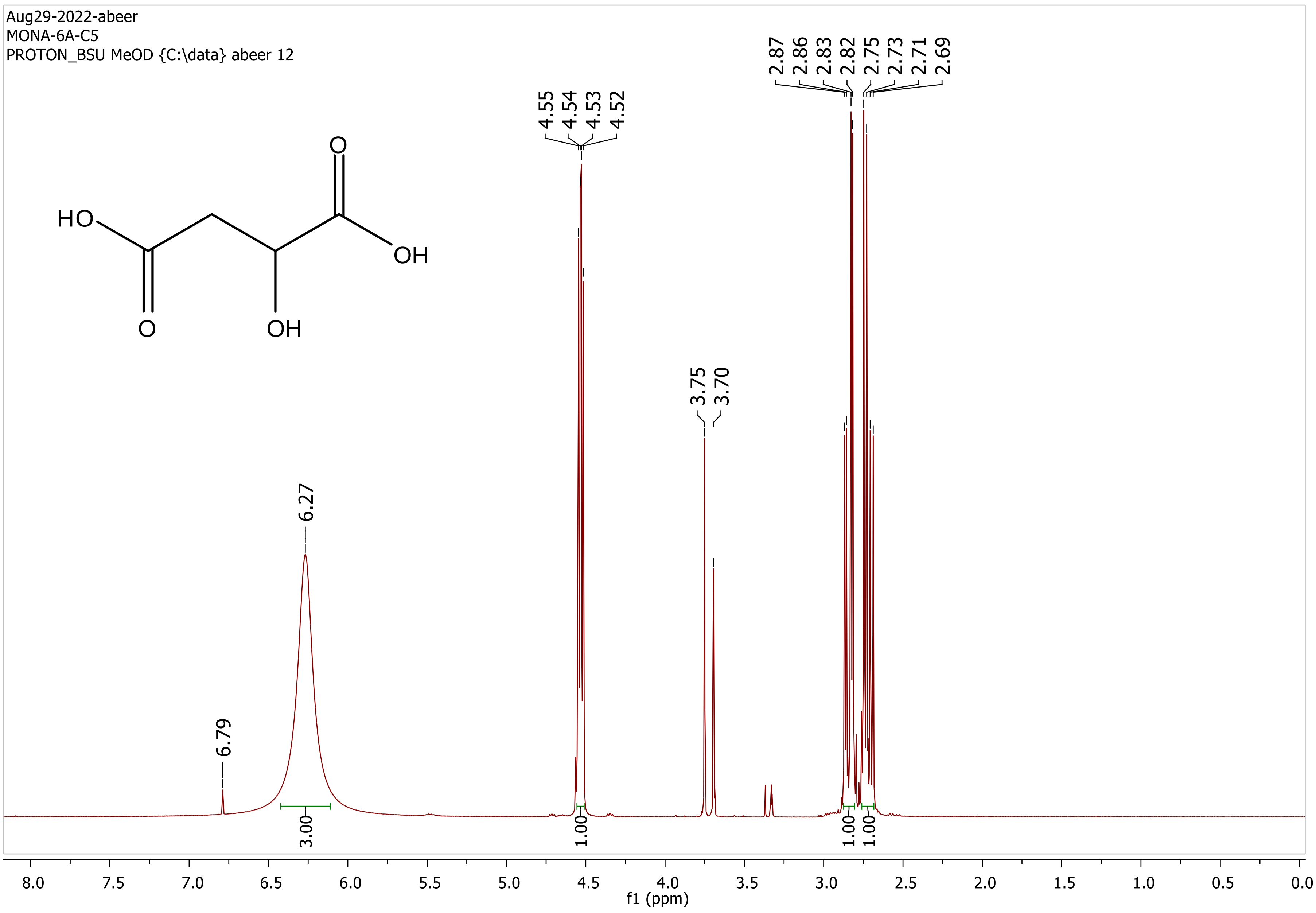

Figure S9: [1] H NMR spectrum of compound C5 Malic acid (400 MHz, CD_3OD)

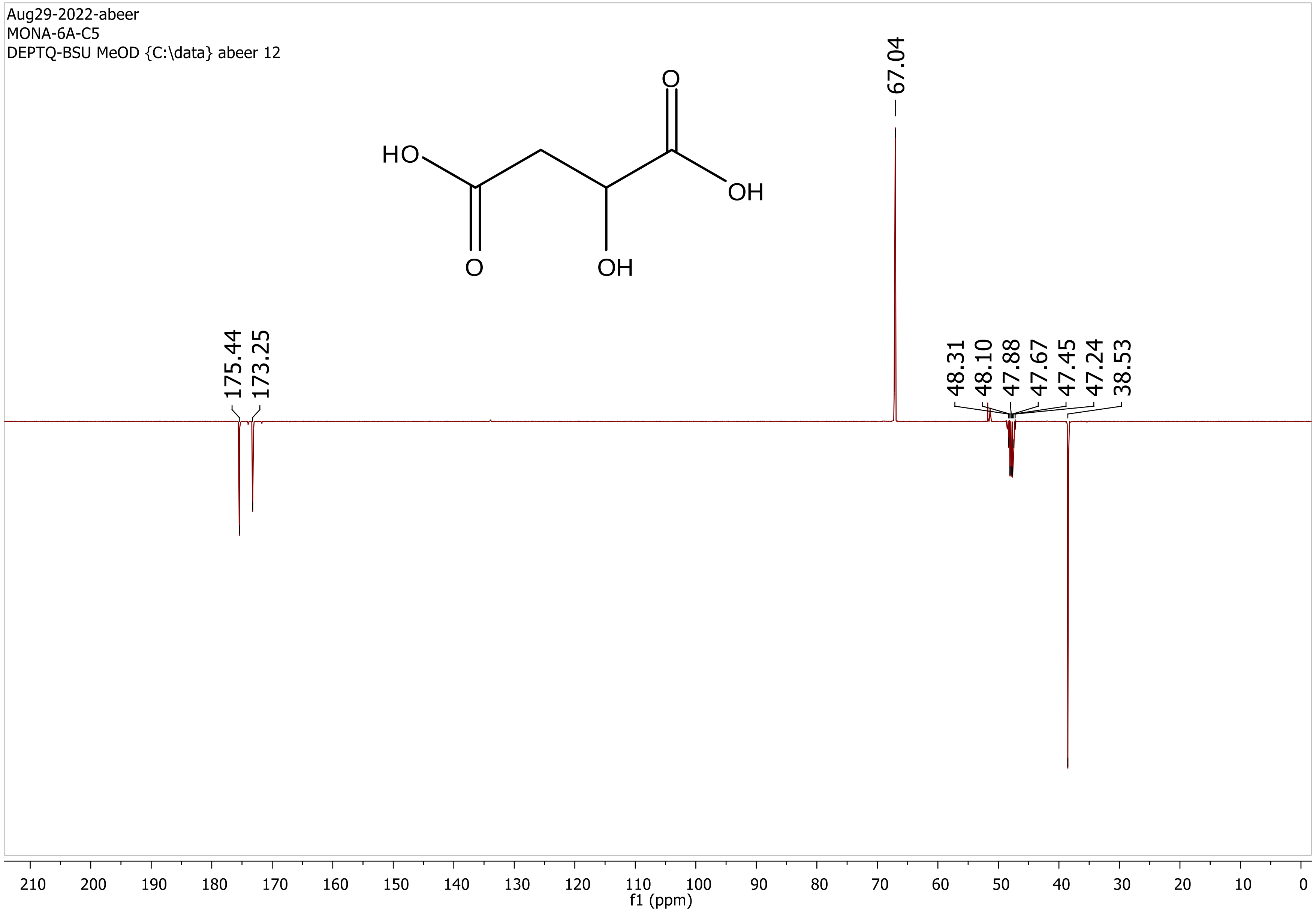

Figure S10: DEPT-Q spectrum of compound C5 Malic acid (100 MHz, CD_3OD)

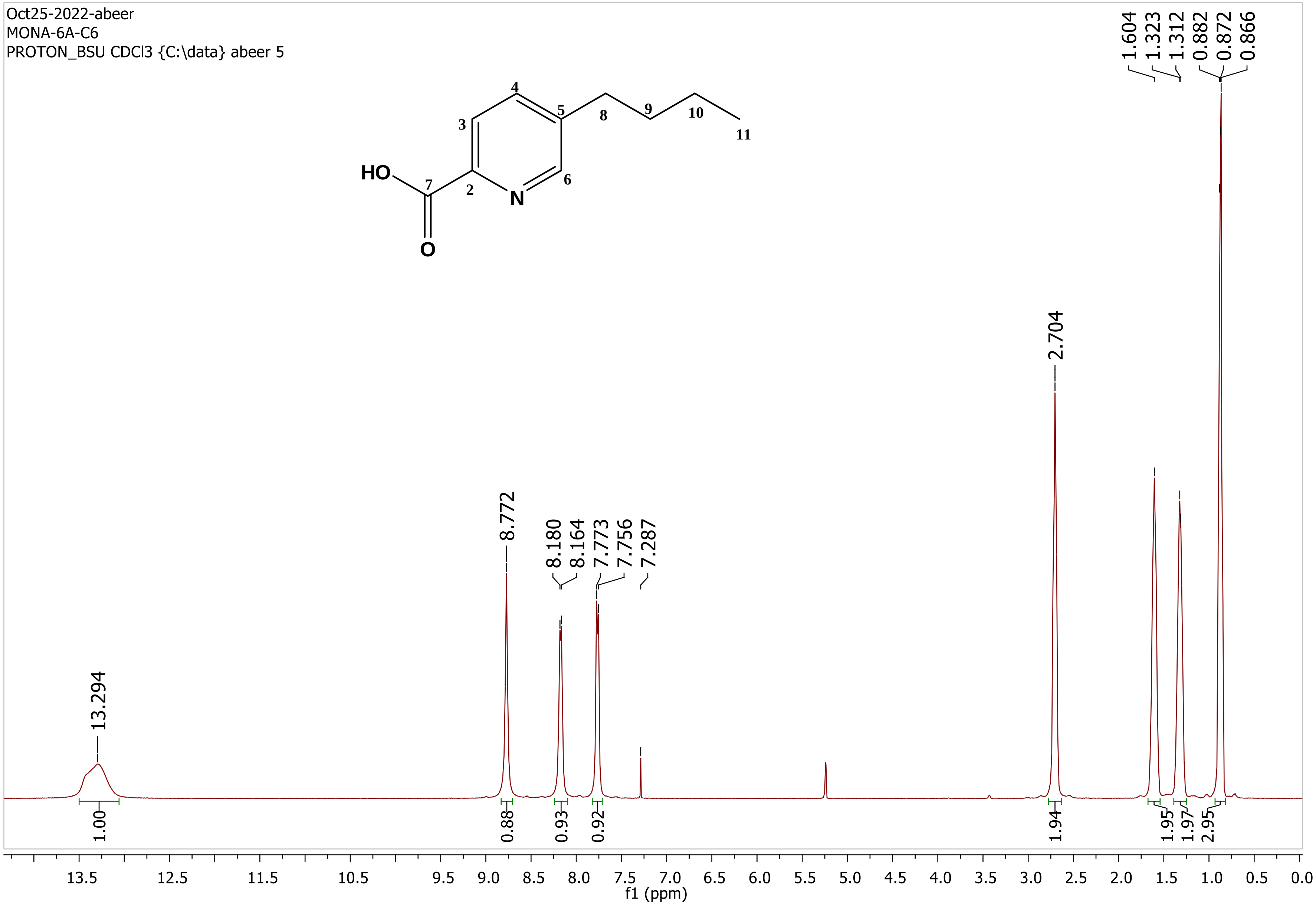

Figure S11: [1] H NMR spectrum of compound C_6 Fusaric acid (400 MHz, CD_3OD)

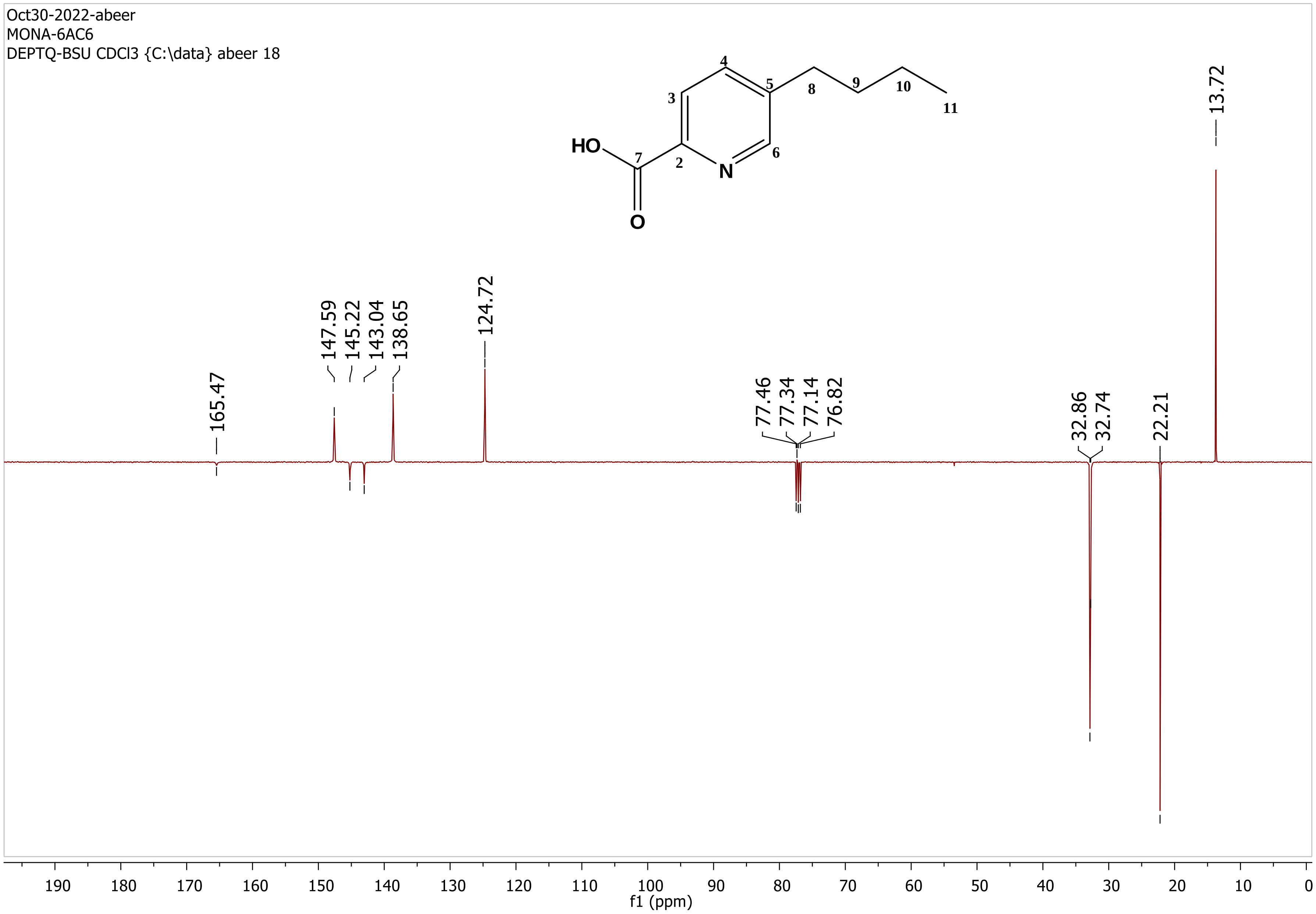

Figure S_{12}: DEPT-Q spectrum of compound C_6 Fusaric acid (100 MHz, CD_3OD)

<u>List of tables:</u>

Table S1: Docking scores of isolated compounds from the endophytic fungus *Fusarium solani* against the enzymes QR2, Hsp90, B-Raf kinase, and pirin

**Compound**	**Docking scores (kcal/mol)**			
	**QR2**	**Hsp90**	**B-Raf kinase**	**Pirin**
4-hydroxybenzaldehyde (**1**)	-4.1	-4.9	-5.3	-5.9
4-hydroxybenzoic acid (**2**)	-4.6	-5.6	-5.8	-6.3
Tyrosol (**3**)	-4.8	-5.1	-5.5	-6.7
Azelaic acid (**4**)	-4.6	-5.0	-5.6	-6.8
Malic acid (**5**)	-4.1	-4.7	-4.4	-5.5
Fusaric acid (**6**)	-5.6	-6.0	-6.1	-7.1
Co-crystallized ligand	-6.3	-9.0	-11.4	-8.1

References

1. Ahmed, E.M., Kassab, A.E., El-Malah, A.A. & Hassan, M.S. Synthesis and biological evaluation of pyridazinone derivatives as selective COX-2 inhibitors and potential anti inflammatory agents. Eur. J. Med. Chem. 171, 25-37 (2019).
2. Amin, E., Elwekeel, A., Alshariedh, N.F., Abdel-Bakky, M.S. & Hassan, M.H. GC-MS Analysis and Bioactivities of the Essential Oil of Suaeda aegyptiaca. Separations. 9, 439-52 (2022).
3. Arung, E.T. et al. Screening of Indonesian plants for tyrosinase inhibitory activity. J. Wood Sci. 51, 520-5 (2005).
4. Khan, N. *et al*. Endophytic Fusarium solani: A rich source of cytotoxic and antimicrobial napthaquinone and aza-anthraquinone derivatives. *Toxicol. Rep*. 5, 970-6 (2018).
5. Falodun, A., Ali, S., Quadir, I.M. and Choudhary, I.M.I. Phytochemical and biological investigation of chloroform and ethyl acetate fractions of *Euphorbia heterophylla* leaf (Euphorbiaceae). *J. Med. Plant Res*. 2, 365-9 (2008).
6. Ghavam-Haghi, F. & Dinani, M.S. Isolation and identification of astragalin and 2-methoxy tyrosol from the bulbs of *Allium paradoxum*. *J. HerbMed Pharmacol*. 6, 114-18 (2017).
7. Jeong-Yong, C. et al. Isolation and identification of azelaic acid and 3, 4-dihydroxybenzoic acid from buckwheat hull as antimicrobial substances. *Food Sci. Biotechnol*. 9, 313-6 (2000).
8. Coutinho, I.D. *et al*. Identification of primary and secondary metabolites and transcriptome profile of soybean tissues during different stages of hypoxia. *Data Br*. 21, 1089-100 (2018).
9. Shi, S. *et al*. Biological activity and chemical composition of the endophytic fungus *Fusarium* sp. TP-G1 obtained from the root of *Dendrobium officinale* Kimura et Migo. *Rec Nat Prod*. 12, 549-56 (2018).
